# Chitinase-3 like-protein-1 promotes glioma progression via the NF-κB signaling pathway and tumor microenvironment reprogramming

**DOI:** 10.7150/thno.75069

**Published:** 2022-10-03

**Authors:** Ting Zhao, Jianming Zeng, Yujie Xu, Zhongping Su, Yulong Chong, Tao Ling, Haozhe Xu, Hui Shi, Minggao Zhu, Qi Mo, Xiaoying Huang, Yingchang Li, Xiaoren Zhang, Hongbin Ni, Qiang You

**Affiliations:** 1 Affiliated Cancer Hospital & Institute, Guangzhou Medical University, Guangzhou 510095, Guangdong, China.; 2 Department of Medical Oncology, Fudan University Shanghai Cancer Center, Shanghai 200032, China.; 3 Faculty of Health Sciences, University of Macau, Taipa, Macau, China.; 4 Department of Biotherapy, Department of Geriatrics, Second Affiliated Hospital of Nanjing Medical University, Nanjing 210011, Jiangsu, China.; 5 Department of Neurosurgery, Nanjing Drum Tower Hospital Clinical College of Nanjing Medical University, Nanjing 210008, Jiangsu, China.; 6 Department of Thoracic Surgery, Shanghai Chest Hospital, Shanghai Jiao Tong University, Shanghai 200030, China.; 7 College of Life Science and Technology, Jinan University, 601 Huangpu Road, Guangzhou 510630, China.; 8 Key Laboratory of Cell Homeostasis and Cancer Research of Guangdong Higher Education Institutes, Guangzhou Medical University, Guangzhou 510182, China.; 9 Center for Cancer and Immunology Research, State Key Laboratory of Respiratory Disease, Guangzhou, China.

**Keywords:** CHI3L1, glioma, NF-κB, ACTN4, tumor microenvironment

## Abstract

**Background:** Chitinase-3-like protein 1 (CHI3L1) is overexpressed in various types of tumors, especially in glioma, and contributes to tumor progression. However, the definite role of CHI3L1 and involved pathway in glioma progression are not completely understood.

**Methods:** CHI3L1 expression in human gliomas and its association with patient survival was determined using enzyme-linked immunosorbent assay, western blot, immunohistochemistry, and public databases. Single-cell RNA-seq was used to characterize the landscape of tumor and myeloid cells. Human proteome microarray assay was applied to identify the binding partners of CHI3L1. Protein-protein interactions were analyzed by co-immunoprecipitation and cellular co-localization. The roles of CHI3L1 in glioma proliferation and invasion were investigated in tumor cell lines by gain- and loss- of function, as well as *in vivo* animal experiments.

**Results:** CHI3L1 was up-regulated in all disease stages of glioma, which was closely related with tumor survival, growth, and invasion. CHI3L1 was primarily expressed in glioma cells, followed by neutrophils. Moreover, glioma cells with high expression of CHI3L1 were significantly enriched in NF-κB pathway. Pseudo-time trajectory analysis revealed a gradual transition from CHI3L1^low^ to CHI3L1^high^ glioma cells, along with the NF-κB pathway gradually reversed from inhibition to activation. Intriguingly, CHI3L1 binds to actinin alpha 4 (ACTN4) and NFKB1, and enhances the NF-κB signaling pathway by promoting the NF-κB subunit nuclear translocation in glioma cells. Further, CHI3L1 were released into the tumor microenvironment (TME) and interacted with CD44 expressed on tumor-associated macrophages to activate AKT pathway, thereby contributing to M2 macrophage polarization. In addition, CHI3L1 positively correlated to the expression of immune checkpoints, such as CD274 (PD-L1) and HAVCR2 (LAG3), which then remodeled the TME to an immunosuppressive phenotype.

**Conclusion:** Our research revealed that CHI3L1 facilitated NF-κB pathway activation within glioma cells and reprogramed the TME, thereby serving as a promising therapeutic target for glioma.

## Introduction

Glioma, a heterogeneous group of primary central nervous system (CNS) tumors, accounts for 80% of all brain tumors 1. Despite numerous aggressive combination therapies including surgery, chemotherapy and radiotherapy, the 5-year overall survival (OS) rate is still poor 2. Hence, identification of key molecules and specific mechanisms associated with the development and occurrence of glioma will aid the accurate diagnosis and treatment.

Nuclear factor (NF)-κB belongs to a family of transcription factors that are involved in viral infection, immune response, cell proliferation and survival, and tumorigenesis 3. Activation of NF-κB pathway in tumor cells has been implicated in the pathogenesis and resistance to chemotherapy, and suppression of NF-κB has been accepted as an attractive therapeutic approach for inhibiting glioma tumor cell proliferation and survival 4. However, the key regulators responsible for NF-κB activation in glioma cells remains unknown and requires further investigation.

In addition to parenchymal cells, immune cells in the tumor microenvironment (TME) undergo substantial reprogramming, thereby acquiring pro-tumor phenotypes to facilitate tumor progression 5, 6. Tumor-associated macrophages (TAMs), the majority of immunocytes in the TME, promote immune escape and tumor progression 7. TAMs in glioma comprise microglia- or monocyte-derived populations, while polarized M2 TAMs contribute to glioma survival and growth by affecting stem cell phenotypes transition 8, angiogenesis 9, and cytokines release 10. TAMs act as therapeutic targets in malignant glioma via the C-C motif chemokine receptor 2/C-C motif chemokine ligand 2 (CCL2) pathway inhibition 11, phenotype transition, and colony stimulating factor 1 receptor blockade 12. Identification of participants that account for M2 TAMs polarization have major implications for glioma therapy.

Chitinase-3-like protein 1 (CHI3L1), a member of the glycoside hydrolase family 18 13, plays crucial roles in oxidative injury, apoptosis, pyroptosis, extracellular matrix regulation, Th1/Th2 inflammatory balance, and parenchymal scarring 14. Non-enzymatic CHI3L1 is highly expressed in various cancer types, such as lung 15, colon, gastric, and breast cancers 16, and is associated with low survival rates of patients with cancer. In particular, up-regulated CHI3L1 and CD44 are considered as markers of mesenchymal (MES) glioma 17 that predominantly manifested NF-κB activation 18. The mechanisms of how CHI3L1 drives NF-κB pathway activation in glioma and participates in TME reprogramming, including TAMs polarization and angiogenesis warrants further investigation.

Here, we analyzed the mRNA-seq data from the Gene Expression Omnibus (GEO), The Cancer Genome Atlas (TCGA), and Chinese Glioma Genome Atlas (CGGA) to explore the expression profile of CHI3L1 in glioma at mRNA level. CHI3L1 protein levels were detected via immunohistochemistry (IHC), enzyme-linked immunosorbent assay (ELISA) and western blot. Spearman correlation analysis was used to explore the association between CHI3L1 expression and immune cells infiltration. Single-cell RNA-seq data were applied to characterize the behavior of tumor cells and delineate the landscape of myeloid cells. The roles of CHI3L1 in glioma survival, proliferation and invasion were further investigated in four glioblastoma (GBM) cell lines and *in vivo* animal experiments.

## Materials and Methods

### Data acquisition and filtration

Glioma transcriptomic profiles and matched clinical information were obtained from the GEO (GSE100675), TCGA, and CGGA, while brain tissues from Genotype-Tissue Expression (GTEx) were chose as controls. A total of 479 samples with clinical information were enrolled in further analysis. Single-cell RNA-seq (GSE117891) that included 70 sample points from 13 patients with glioma were obtained from GEO which was publicly available online. For the comparison of gene expression among different human glioma cell lines, we applied the “Expression 21Q3 Public” file downloaded from the Cancer Cell Line Encyclopedia (CCLE) website.

### Absolute immune cell infiltration and immune subsets

The estimation of the total immune cell infiltration within each glioma sample and the immune cell subsets was determined by Cibersort using the LM22 gene set on the Cibersort website 19. The association between the gene expression and immune infiltrates was performed by Spearman correlation analysis.

### Human serum and samples

We collected serum samples from 69 patients with staged glioma and 25 healthy volunteers, and 41 pairs of clinical diagnosed glioma and peritumoral tissues from the Department of Neurosurgery of The Affiliated Drum Tower Hospital of Nanjing University Medical School (Nanjing, Jiangsu, China) between January 2020 and September 2021 as approved by the Clinical Ethics Committee.

### Enzyme-linked immunosorbent assay (ELISA)

Serum levels of CHI3L1 in human were measured by ELISA kits (Cat. #EK1164-96, Multisciences, Hangzhou, China) according to the manufacturer's protocol.

### Histological analysis

Human glioma and paired normal brain tissues were fixed in 10% formalin, embedded in paraffin and cut into 4 μm thick sections. Sectioned tissues were further performed for Hematoxylin and eosin (H&E) staining and IHC staining with anti-CHI3L1 (Cat. #ab77528, Abcam). For immunofluorescence (IF), frozen brain sections (6 μm) were incubated with anti-CHI3L1 (Cat. #ab77528, Abcam) and anti-NF-κB p65 (Cat. #6956, CST) antibody. Meanwhile, we incubated frozen sections with anti-human CD163 (Cat. #4332578, Invitrogen) and anti-human CD206 (Cat. #4306524, Invitrogen) antibodies to evaluate M2 macrophage infiltration.

### Human proteome microarrays

We utilized a human proteome microarray from Wayen Biotechnologies (Shanghai), Inc. The recombinant human CHI3L1 (rhCHI3L1) was purchased from Sino Biological lnc (Human, Cat. #11227-H08H). The proteome microarray assay was performed as published previously 20.

### GBM cell lines culture and treatment

Human GBM cell lines (U87MG, U251MG, U118MG and A172) authenticated by STR profiling were purchased from Procell Life Science & Technology Co., Ltd (Wuhan, China) and Cell Bank of Chinese Academy of Sciences (Shanghai, China). Cells were cultured in Dulbecco's modified Eagle's medium (DMEM) (Gibco, USA) supplemented with 10% fetal bovine serum (FBS) (Gibco, USA), and incubated in a humidified incubator at 37 °C with 5% CO_2_. The glioma cells were treated with TNFɑ (50 ng/mL or 200 ng/mL) (Cat. #GMP-10602-HNAE, Sino Biological lnc) to study the effect of CHI3L1 on NF-κB pathway activation after shRNAs transinfection. All cells were negative for mycoplasma.

### Bone marrow derived macrophages (BMDM) isolation and culture

BMDMs were isolated and cultured for seven days in the presence of recombinant murine M-CSF (10 ng/mL) (Cat. # 51112-MNAH, Sino Biological lnc) for further studies.

### shRNA preparation and CHI3L1 knock-down

Scrambled and human CHI3L1 shRNA plasmids were purchased from GenePharma (Shanghai, China) and performed according to the manufacturer's instructions. Sequences for control and target specific shRNAs are as following: control shRNA, 5'- TTC TCC GAA CGT GTC ACG T-3'; CHI3L1 shRNA-1, 5'-CAA GGA AAT GAA GGC CGA ATT-3'; CHI3L1 shRNA-2, 5'-TAG CAT CAT GAC CTA CGA TTT-3'. U87MG and A172 cell lines were transfected with shRNAs using Lipo3000 Transfection Reagent (Cat. #L3000150, Invitrogen) and western bolt was applied to assess the transfection and knockdown efficiencies.

### Lentivirus

The lentiviral shRNA vector (hU6-MCS-CBh-gcGFP-IRES-puromycin) targeting human Chi3L1 was constructed and the particles were produced by Shanghai Genechem Co.,Ltd. (Shanghai, China). The shRNA targeting sequence is CAA GGA AAT GAA GGC CGA ATT. The lentiviral expression vector pLV2-TRE3GS-CHI3L1 (human)-TetOne-Puro was constructed by MiaoLing Plasmid Platform (Wuhai, China). The lentiviruses were packaged using recombinant lentiviral vector plasmid (pLV2-TRE3GS-CHI3L1, 12 μg), packaging plasmid (psPAX2, 9 μg) and envelope plasmid (pMD2G, 3 μg) for 4 × 10^6^ of HEK293 cells in one 10 cm dish. Briefly, the three plasmids were resuspended in 1.5 mL Opti-MEM, and mixed with 1.5 mL Opti-MEM containing 60 μL polyetherimide (PEI) for 20 min at room temperature. Then, the mixture were added to HEK293 cells dropwise. The culture medium was changed to fresh medium after 8 h. Subsequently, the culture medium containing lentivirus was collected after 24 h and 48 h. Then, U251 cells were infected with lentiviruses and screened by puromycin (2 μg/mL) to obtain stable cell lines. For inducible CHI3L1 expression, doxycycline (Dox) (1 μg/mL) was added to the resultant U251 cells for 24 h.

### Subcutaneous tumor growth assay

U87MG cells stably infected with lenti-shCHI3L1 or lenti-shControl (3 × 10^6^ cells in 0.1 mL PBS) were subcutaneously injected into nude mice (6-8 weeks of age, n = 6 per group) (GemPharmatech, Nanjing, China) per site in each mouse. Tumors were measured every other day using calipers and the volume determined using the formula: V = (S^2^ × L)/2, where V is the volume, S is the shortest diameter, and L is the longest diameter. The mice were euthanized on day 16, and the tumors were measured and photographed.

### Immunofluorescence (IF) staining

For macrophage polarization studies, macrophages derived from THP1 cells induced by phorbol 12-myristate 13-acetate (PMA) (10 ng/mL) (Cat. #P8139, Sigma) for 24 h, as well as differentiated BMDM, were subsequently incubated with recombinant CHI3L1 (500 ng/mL) (Human, Cat. #11227-H08H; Murine, Cat. #50929-M08H, Sino Biological lnc) in the presence of recombinant IL-4 (100 ng/mL) (Human, Cat. #200-04; Murine, Cat. #214-14, PeproTech) as a positive control. Macrophages polarization was assessed by IF staining using CD163 and CD206 to identify M2 macrophages.

### Nuclear and cytoplasmic protein extraction

Nuclear and cytoplasmic proteins were isolated for further western blot detection using Nuclear and Cytoplasmic Protein Extraction Kit (Cat: #P0028, Beyotime) according to the manufacturer's instructions.

### Co-immunoprecipitation (Co-IP)

U87MG and A172 cells were harvested and lysed using IP buffer (20 mM Tris at pH 7.5, 150 mM NaCl, 1% TritonX-100, 1 mM EDTA, and protease inhibitors) on ice. Cell extracts were incubated with anti-rabbit IgG (0.5 μg) or anti-CHI3L1 antibody (Cat. #77528, Abcam) (0.5 μg) overnight at 4 °C on a rotator, followed by addition of protein G agarose beads for 2 h at 4 °C. Next morning, the complexes were resuspended and then subjected to 10% SDS-PAGE for further immunoblotting.

### Western Blot

Tissues and cells were lysed in lysis buffer and electrophoresed in 10% SDS-PAGE gels, and then probed with specific antibodies listed in Supplementary Table 1.

### NF-kB dual-luciferase activity reporter assay

HEK293 cells were seeded in 12-well plates and transfected on the following day by PEI 40K Transfection Reagent (Cat#: G1802, Wuhan Servicebio Technology Co., Ltd, China) according to the manufacturer's instructions. In brief, the cells were transfected with the NF-kB firefly luciferase activity reporter plasmid in combination with either empty vector, or CHI3L1, or ACTN4, or the combination of CHI3L1 and ACTN4 plasmids. To normalize for transfection efficiency, pRL-TK (Renilla luciferase) reporter plasmid was added to each transfection. Then, the cells were treated with TNFα (20 ng/mL) for 24 h. Subsequently, the cells were harvested and measured luciferase activity using a Dual Luciferase Reporter Assay Kit (Cat#: DL101-01, Vazyme, Nanjing, China). Firefly luciferase activities were normalized on the basis of Renilla luciferase activities.

### Flow cytometry

HA-hCHI3L1 plasmid was constructed by Jiangsu Genecefe Biotechnology Co., Ltd (Wuxi, China). The plasmids were transfected into U251MG or U118MG cells by PEI 40K Transfection Reagent. Forty-eight hours after transfection, the cells were harvested, blocked, and stained by PE anti-human CD274 (PD-L1, B7-H1) antibody (Cat#: 12-5983-42, clone: MIH1, Thermo Fisher Scientific). Then, flow cytometry was performed using BD FACSC II flow cytometer, and the data were analyzed by Flow-Jo software, v10.0.

### Quantitative real-time PCR (qRT-PCR)

Total RNA was extracted with RNA Isolation Kit (Cat. #RC112-01, Vazyme), and 1 μg of RNA was immediately applied for reverse transcription (Cat. #R323-01, Vazyme). Gene expression was normalized to GAPDH, and the primer sequences were available in Supplementary Table 2.

### Colony formation assay

Approximately 1000 glioma cells were seeded in a 6-well plate and were grown for 14 days. Cell colonies (≥50 cells) were fixed and then stained with Giemsa solution to facilitate counting.

### EdU cell proliferation assay

EdU incorporation was performed using BeyoClick™ EdU-488 kit (Cat. #C0071S, Beyotime) per manufacturer's instructions after transfected with control or CHI3L1 shRNA to evaluate the proliferative activity of U87MG and A172 cells.

### Wound healing and migration assays

U87MG and A172 cells transfected with control or CHI3L1 shRNA were cultured in Two-well culture-inserts (Cat. #80209, ibidi) in a 24-well cell culture plate. Remove the culture-inserts and then fill with serum-free medium, and the cell-free gaps were continuously observed for 12 h under a microscope. FLAG-hACTN4 plasmid was purchased from (Cat#: HG10971-CF) (Sino Biological lnc. Beijing, China).

### Cell proliferation assay

The proliferation of glioma cells were evaluated by Cell Counting Kit-8 (CCK8) assay. Cells were seeded at 1 × 10^4^ cells per well in serum-free medium in a 24-well plate. The absorbance at 450 nm of each well was measured at 0, 24, 48, 72 and 96 hours.

### Statistical analysis

R version 4.0.4 software was used for raw high-throughput RNA sequencing and single cell RNA sequencing data analysis. Fiji was utilized for image analysis, and GraphPad Prism 9.0 was applied for quantification and statistical analysis. Survival analysis was performed by log-rank test in Kaplan-Meier curves. For comparisons, Student's t-test and Wilcoxon test were applied to compare the differences between groups; one-way ANOVA was used for comparisons among three or more groups.

## Results

### CHI3L1 is over-expressed in glioma and associated with worse OS

**Figure 1:**
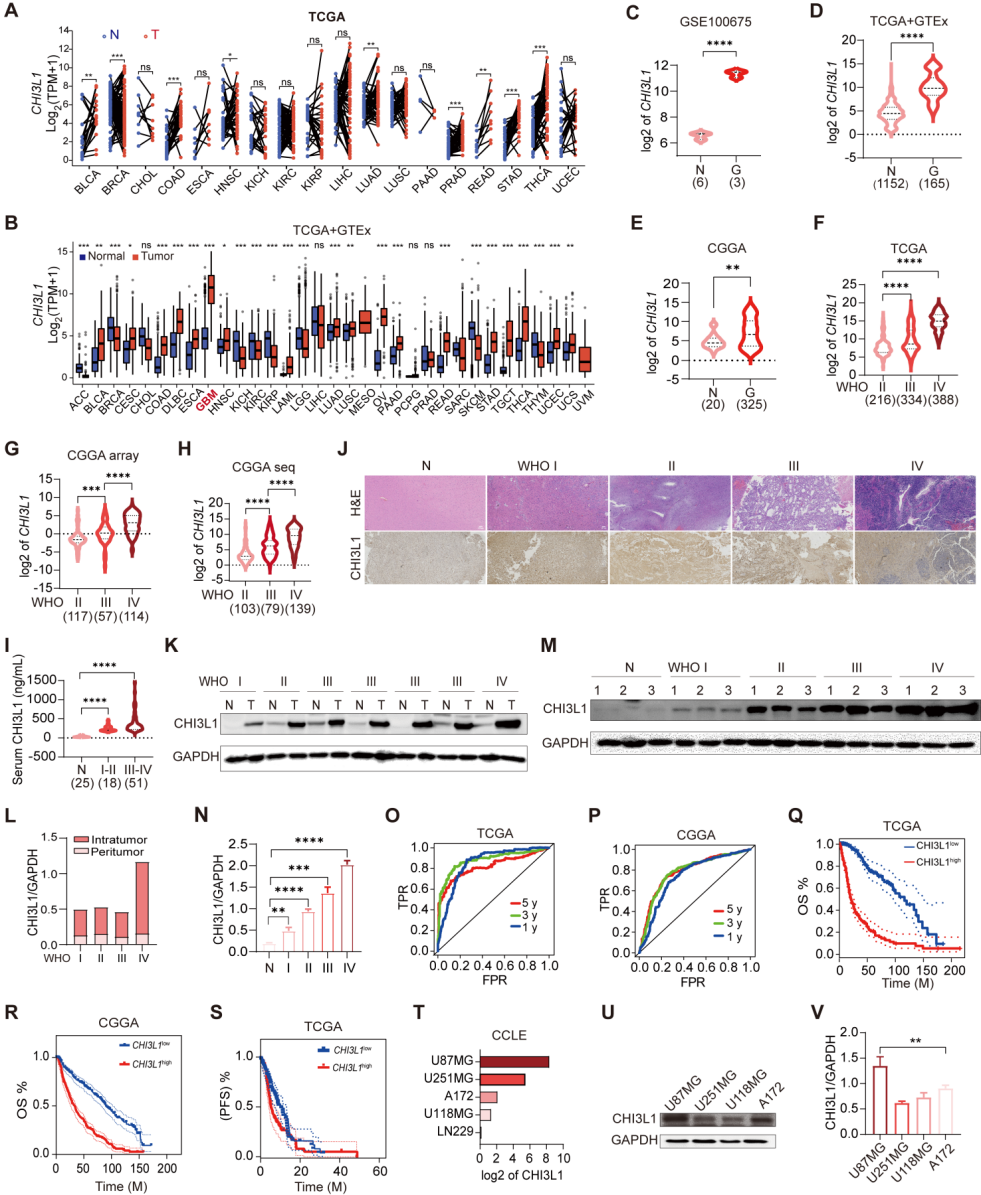
** CHI3L1 expression is correlated with the malignancy and OS of glioma**. **(A-B)** The mRNA levels of CHI3L1 in 33 cancer types and paired normal tissues in TCGA. **(C-E)** CHI3L1 expression levels in normal and glioma tissues in GEO, TCGA and CGGA. **(F-H)** CHI3L1 expression levels in staged gliomas in GEO, TCGA and CGGA. **(G)** CGGA mRNA_array_301, **(H)** CGGA mRNAseq_325. **(I)** Serum levels of CHI3L1 in patients with glioma. **(J)** Representative pictures of H&E staining and IHC analysis of CHI3L1 expression levels in glioma and paired normal tissues; Scale bars represent 100 µm. **(K-L)** Western blot and quantitative analysis of CHI3L1 expression in seven pairs of glioma with different grades and paired normal tissues. **(M-N)** Western blot and quantitative analysis of CHI3L1 expression in normal tissues and staged gliomas. **(O-P)** ROC curve exhibited the sensitivity and specificity of CHI3L1 to predict glioma in TCGA and CGGA databases. **(O)** 5 y (AUC = 0.819), 3 y (AUC = 0.881), 1 y (AUC = 0.852). **(P)** 5 y (AUC = 0.784), 3 y (AUC = 0.782), 1 y (AUC = 0.741), y: year. TPR: true positive rate. FPR: False positive rate. **(Q-S)** Survival benefits between CHI3L1^high^ and CHI3L1^low^ groups (median was the cut-off to identify high and low groups) were assessed via Kaplan-Meier analysis using both the Log-rank and Wilcoxon-Breslow tests. **(Q)** Log-rank p = 0, HR (high) = 6.4, p (HR) = 0, n (high) = 338, n (low) = 337.** (R)** Log-rank p < 0.0001, Wilcoxon p < 0.0001. **(S)** Log-rank p = 0.0692, Wilcoxon p = 0.0243. **(T)** The mRNA expression profile of CHI3L1 in glioma cell lines from the CCLE. **(U-V)** Comparison of CHI3L1 expression levels in glioma cell lines analyzed by one-way analysis of variance (ANOVA). N: normal. G: glioma. T: tumors. OS: overall survival. PFS: progression-free survival. M: months. Student's t test, *p < 0.05; **p < 0.01; ****p < 0.0001.

We analyzed TCGA pan-cancer and GTEx (15776 samples in total) mRNA data to compare the expression levels of CHI3L1 in 33 tumor types and paired normal tissues. CHI3L1 was found to be over-expressed in cancer lesions than in the normal adjacent tissues (Figure 1A). GBM is the type of tumor with the highest expression of CHI3L1 compared to other tumor types (Figure 1B). To gain more insight into the relationship between CHI3L1 expression and glioma phenotype, we extracted mRNA microarray or sequencing cases with detailed clinical information from GEO, TCGA and CGGA databases. CHI3L1 was over-expressed in tumors compared to normal regions in patients with glioma (Figure 1C-E), and the expression levels of CHI3L1 increased with increasing grades of glioma (Figure 1F-H). The mRNA levels of *CHI3L1* are positively correlated with the WHO grade, IDH status, and TCGA subtypes of gliomas in TCGA and CGGA datasets (Supplementary Table 3). Moreover, the serum levels of CHI3L1 in patients with glioma were higher than those of healthy controls detected by ELISA (Figure 1I). To better clarify the expression patterns of CHI3L1 within giloma, we collected peritumor and intratumor tissues by image-guided multiregional sampling (Supplementary Figure 1). IHC showed that CHI3L1 was enriched in staged gliomas, especially in high grade gliomas (HGG) (WHO III-IV) (Figure 1J). Western blot also confirmed that CHI3L1 was significantly up-regulated in tumor sites than in peritumor cores (Figure 1K-L), and its expression increased along with the increase of WHO grade of giloma (Figure 1M-N).

As CHI3L1 had been proved to be a biomarker of inflammatory and neoplastic disease 21, we plotted ROC curves and calculated the area under the curve (AUC) to estimate the diagnostic performance of CHI3L1 in glioma in TCGA and CGGA, and the AUC value (one year) were up to 0.852 and 0.741, respectively (Figure 1O-P). Kaplan-Meier analysis revealed that higher levels of CHI3L1 were associated with worse progression-free survival (PFS) and OS rate in patients with glioma (Figure 1Q-S). Likewise, univariate Cox regression analysis revealed a significant correlation between CHI3L1 expression and clinical outcomes in the CGGA_mRNA-array_301 and CGGA_mRNAseq_693 cohorts (Supplementary Table 4).

For the gene expression profiles in GBM cell lines, data of the five GBM cell lines were obtained from the CCLE website to compare the CHI3L1 expression (Figure 1T). With regard to protein level, western blot documented that CHI3L1 was higher in U87MG and A172 cells than that of U251MG and U118MG cells (Figure 1U-V). Taken together, CHI3L1 was highly expressed in tumor sites and was significantly correlated with poor survival in patients with glioma.

### CHI3L1 expression is correlated to the activation of the NF-κB pathway in glioma

**Figure 2:**
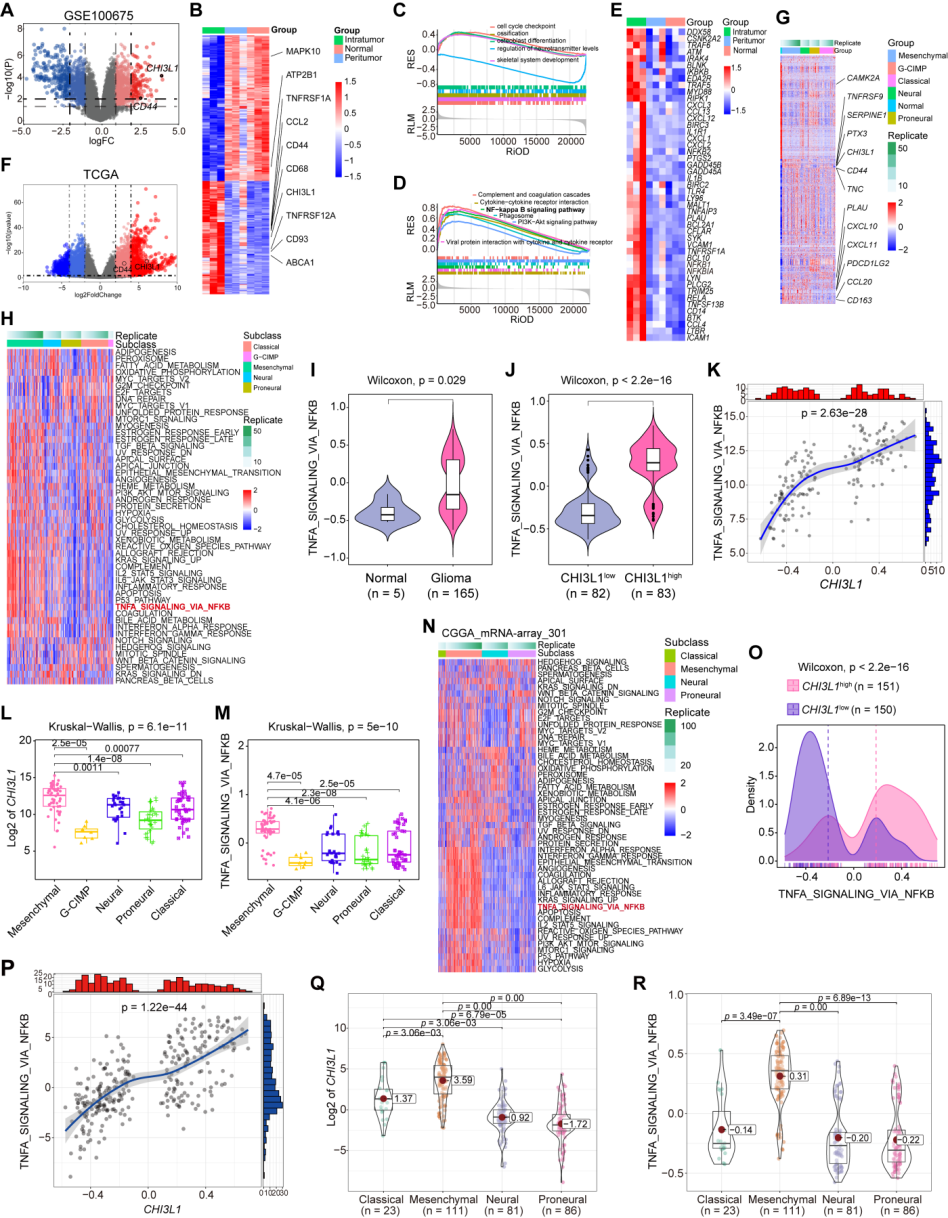
** CHI3L1 expression is associated with NF-κB pathway activation in glioma. (A-B)** Volcano plot and heatmap of differentially expressed genes (DEGs) in GSE100675 dataset. **(C-D)** GO and GSEA based on DEGs identified in GSE100675. **(E)** Heatmap of genes targeting the NF-κB pathway in normal and tumors. **(F-G)** Volcano plot and heatmap of DEGs in TCGA glioma cohort. **(H)** Heatmap of 50 hallmark gene sets performed by GSVA in TCGA. **(I-J)** Enrichment score of TNFɑ NF-κB pathway among normal and tumor, as well as among CHI3L1^high^ and CHI3L1^low^ groups in TCGA, Wilcoxon test. **(K)** Spearman correlation analysis between the mRNA expression of CHI3L1 and NF-κB pathway enrichment score in TCGA. *t*_student_ (163) = 13.48, r_Pearson_ = 0.73, CI_95%_ [0.64, 0.79], n_pairs_ = 165. log_e_ (BF_01_) = -57.89, 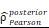
= 0.72, 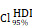 = [0.64, 0.79], 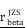 = 1.41.** (L-M)** CHI3L1 expression and TNFɑ NF-κB pathway enrichment score between the five subclasses of glioma in TCGA, Kruskal-wallis test. **(N)** Heatmap of 50 hallmark gene sets by GSVA in CGGA mRNA-array 301 dataset. **(O)** Density plot showed the TNFɑ NF-κB pathway enrichment score between CHI3L1^high^ and CHI3L1^low^ groups in CGGA, Wilcoxon test. **(P)** Spearman correlation analysis between CHI3L1 expression and NF-κB pathway enrichment score in CGGA. *t*_student_ (299) = 16.69, r_Pearson_ = 0.69, CI_95%_ [0.63, 0.75], n_pairs_ = 301. log_e_ (BF_01_) = -95.06, 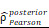
= 0.69, 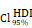 = [0.63, 0.75], 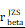 = 1.41.** (Q-R)** Violin plot showed the expression of CHI3L1 and TNFɑ NF-κB pathway enrichment score between the four subclasses of glioma in CGGA, Kruskal-wallis test. **(Q)** F_welch_ (3, 90.33) = 101.56, p = 7.72e-29, CI_95%_ [0.69, 1.00], n_obs_ = 301. **(R)** F_welch_ (3, 89.73) = 103.07, p = 5.70e-29, CI_95%_ [0.70, 1.00], n_obs_ = 301. RES: Ranked Enrichment Score. RLM: Ranked List Metric. RiOD: Rank in Ordered Dataset.

Aberrant activation of cachexia signaling pathway contributes to glioma progression 17; therefore, we analyzed the GEO, TCGA and CGGA cohorts to identify the activated pathways in glioma. In GSE100675, 625 up- and 803 down-regulated genes (logFC = 1) were visualized in the volcano plot and heatmap, and CHI3L1 was markedly up-regulated in tumors (Figure 2A-B). To further analyze this signature, we performed Gene Ontology (GO) and Gene Set Enrichment Analysis (GSEA), which identified cell cycle checkpoint and NF-κB signaling pathway as the most significantly enriched functional term and pathway, respectively (Figure 2C-D). The target genes of the NF-κB pathway in normal, peritumor, and intratumor tissues were illustrated in the heatmap (Figure 2E).

In TCGA cohort, we identified 2546 up- and 1647 down-regulated genes in patients with glioma (log2FordChange = 3). CHI3L1 was plotted as an over-expressed gene on the volcano plot and heatmap in TCGA cohorts (Figure 2F-G). To better characterize the relationship between CHI3L1 expression and NF-κB pathway enrichment, we applied Gene Set Variation Analysis (GSVA) of the Hallmark gene set to obtain quantitative analysis for specific pathway. Among the five TCGA subtypes (G-CIMP, MES, classical (CL), neural (NL), and proneural (PL), the distribution of 50 signaling pathways enrichment analysis were visualized in the heatmap (Figure 2H). TNFɑ signaling via the NF-κB pathway was significantly enriched in tumor sites (Figure 2I), and the enrichment score of NF-κB pathway was higher in the CHI3L1^high^ group than that of the CHI3L1^low^ group (patients were divided into CHI3L1^high^ and CHI3L1^low^ groups based on a median cut-off of the CHI3L1 expression) (Figure 2J). Interestingly, the enrichment score of the NF-κB pathway was positively correlated with the expression of CHI3L1 in glioma (Figure 2K), especially in MES glioma which had the highest expression of CHI3L1 (Figure 2L-M).

In the GSVA of the CGGA mRNA-array_301 dataset, patients in CHI3L1^high^ group exhibited a higher enrichment score than that of the CHI3L1^low^ group (Figure 2O), which was consistent with the result of TCGA. Likewise, we observed a significant positive correlation between the enrichment score of the NF-κB pathway and CHI3L1 expression in CGGA (Figure 2P). MES glioma possessed the highest enrichment score of NF-κB pathway with the highest mRNA expression of CHI3L1 (Figure 2Q-R). In conclusion, comprehensive analysis based on transcriptome data suggested that CHI3L1 promoted the activation of the NF-κB pathway to serve as an accomplice during the progression of glioma.

### CHI3L1^high^-specific glioma cells dominantly drive the NF-κB pathway activation in glioma at the single-cell level

**Figure 3:**
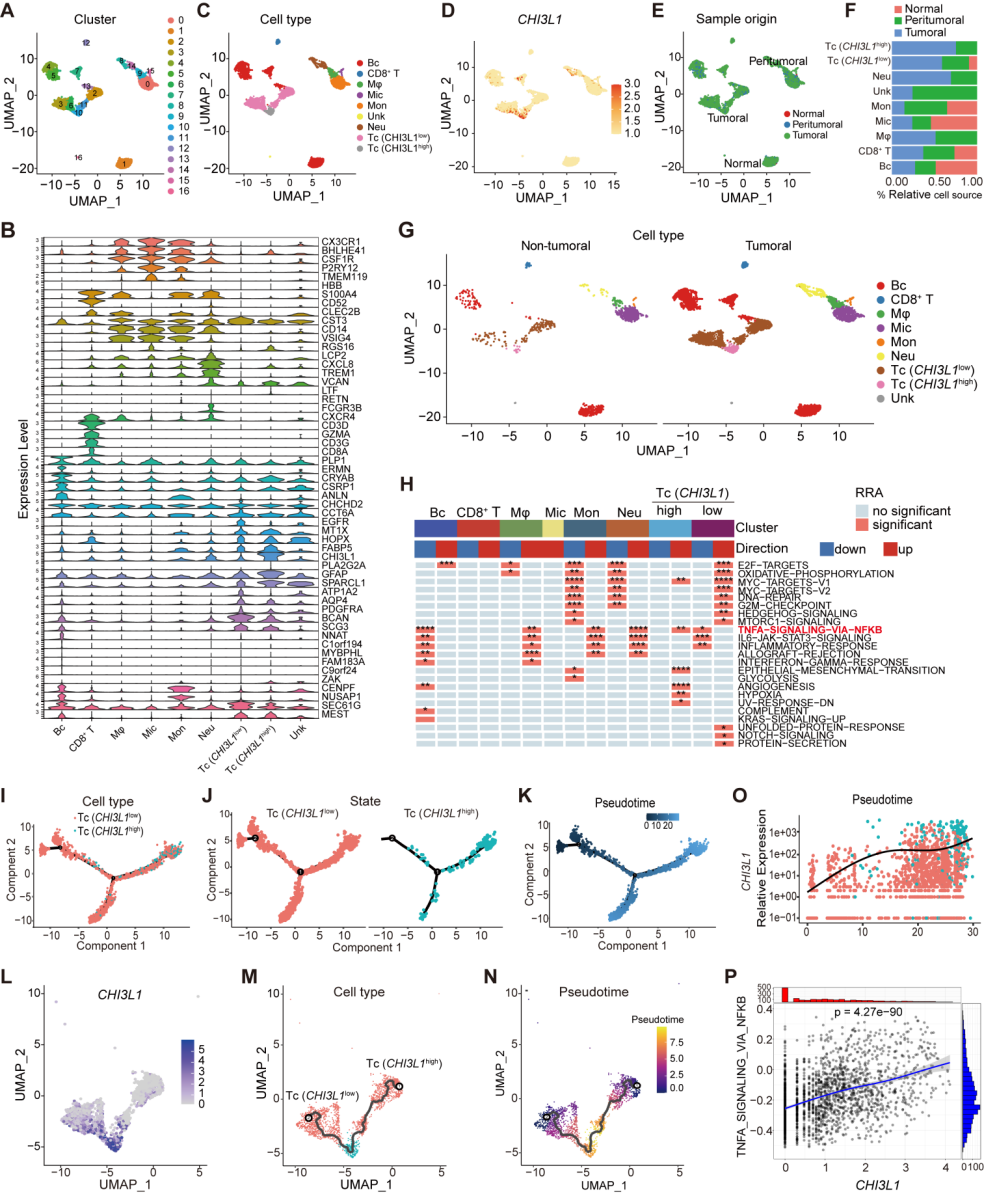
** CHI3L1^high^ specific glioma cells dominantly drive the activation of the NF-κB pathway**. UMAP plot of total cells from patients with staged gliomas, with each cell color coded for cluster **(A)**, cell type **(C)**, and sample origin **(E-F)**. **(B)** Violin plot showed marker genes for cell annotation. **(D)** CHI3L1 expression profiles in all cells in the UMAP plot. **(G)** UMAP plot of all cells in tumoral and non-tumoral regions coded for cell type. **(H)** Heatmap of 50 hallmark gene sets in eight identified cell clusters using singscore. **(I-K)** Unsupervised transcriptional trajectory of CHI3L1^high^ and CHI3L1^low^ glioma cells, coded by cell type, state, and pseudotime. **(L)** UMAP dot plot of CHI3L1 expression levels in gliomas. **(M-N)** Pseudotime trajectory of CHI3L1^high^ and CHI3L1^low^ glioma cells in the UMAP dot plot. **(O)** Dynamic changes in CHI3L1 expression levels in all glioma cell types and across pseudotime. **(P)** The correlation between expression of CHI3L1 and NF-κB pathway enrichment score. *t*_student_ (1954) = 21.22, *r*_Pearson_ = 0.43, CI_95%_ [0.40, 0.47], n_pairs_ = 1956. log_e_ (BF_01_) = -198.95, 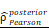
= 0.43, 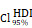 = [0.40, 0.47], 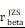 = 1.41. Bc: brain cell, Mφ: Macrophage, Mic: Microglia, Mon: Monocyte, Unk: Unknown, Neu: Neutrophil, Tc: Tumor cell. *p < 0.05; **p < 0.01; ***p < 0.001; ****p < 0.0001.

We analyzed the multiregional glioma single-cell transcriptomic sequencing data (GSE117891) 22 from the GEO database to further understand the mechanisms by which cells regulated NF-κB pathway activation in glioma. Total cell populations from 70 sample points of 13 patients, which consisted of three low grade gliomas (LGG) and 10 GBM cases (GS15 was excluded for lung cancer with brain metastasis), were included in the analysis (total 7160 cells: normal cells, 64; peritumoral cells, 1200; tumoral cells, 5896). Uniform manifold approximation and projection (UMAP) analysis of the total cell populations identified 17 sub-clusters (~sc-0-16, resolution = 1) (Figure 3A), including immune cells (myeloid and T cells: sc-0, 8, -9, and -14; sc-12, respectively), brain cells (sc-1, -4, -5, -7, -13), tumor cells (sc-2, -3, -6, -10, and -11), and microglia (sc-15), and the sc-16 population was too small to allow meaningful definition, segregated primarily by their lineage identities (Figure 3C). Marker genes of subclusters of cells are presented in the violin plot (Figure 3B). Surprisingly, CHI3L1 was preferentially expressed in glioma cells, followed by neutrophils (Figure 3D). We identified two different types of tumor cells that showed high levels of glioma markers: *CST3, CCT6A, EGFR, MT1X, HOPX, FABP5, CHI3L1, GFAP, SPARCL1, ATP1A2, AQP4, PDGFRA, BCAN* and* SCG3* 17. Among them, cluster 10 was defined as CHI3L1^high^ tumor cells with high expression of CHI3L1, while others were attributed to the CHI3L1^low^ group (Figure 3B-D). In the TME, there was a significant increase in neutrophil and a sharp decrease in monocyte compared with peritumoral cores (Figure 3E-F). For ease of comparison, we merged cell populations from normal and peritumoral sites into a new group, which was defined as non-tumoral (Figure 3G).

We first focused on the role of CHI3L1 expression on NF-κB pathway activation in malignant cells. GSVA indicated that the CHI3L1^high^ glioma cells were significantly enriched in TNFɑ signaling via the NF-κB pathway, while the CHI3L1^low^ glioma cells exhibited inhibited NF-κB pathway enrichment (Figure 3H). We then conducted a pseudo-time analysis (monocle 2) to order tumor cells in pseudo-time to infer their developmental trajectories, and pseudo-time trajectories revealed a gradual transition from CHI3L1^low^ to CHI3L1^high^ tumor cells (Figure 3I-N). We also observed a positive correlation between CHI3L1 expression and NF-κB signaling pathway activation at single-cell level (Figure 3O-P), which implied that CHI3L1 played an indispensable role in the activation of the NF-κB signaling pathway in glioma.

### CHI3L1 binds to ACTN4 and NFKB1, and enhances the NF-κB signaling pathway by promoting the NF-κB subunit nuclear translocation

**Figure 4:**
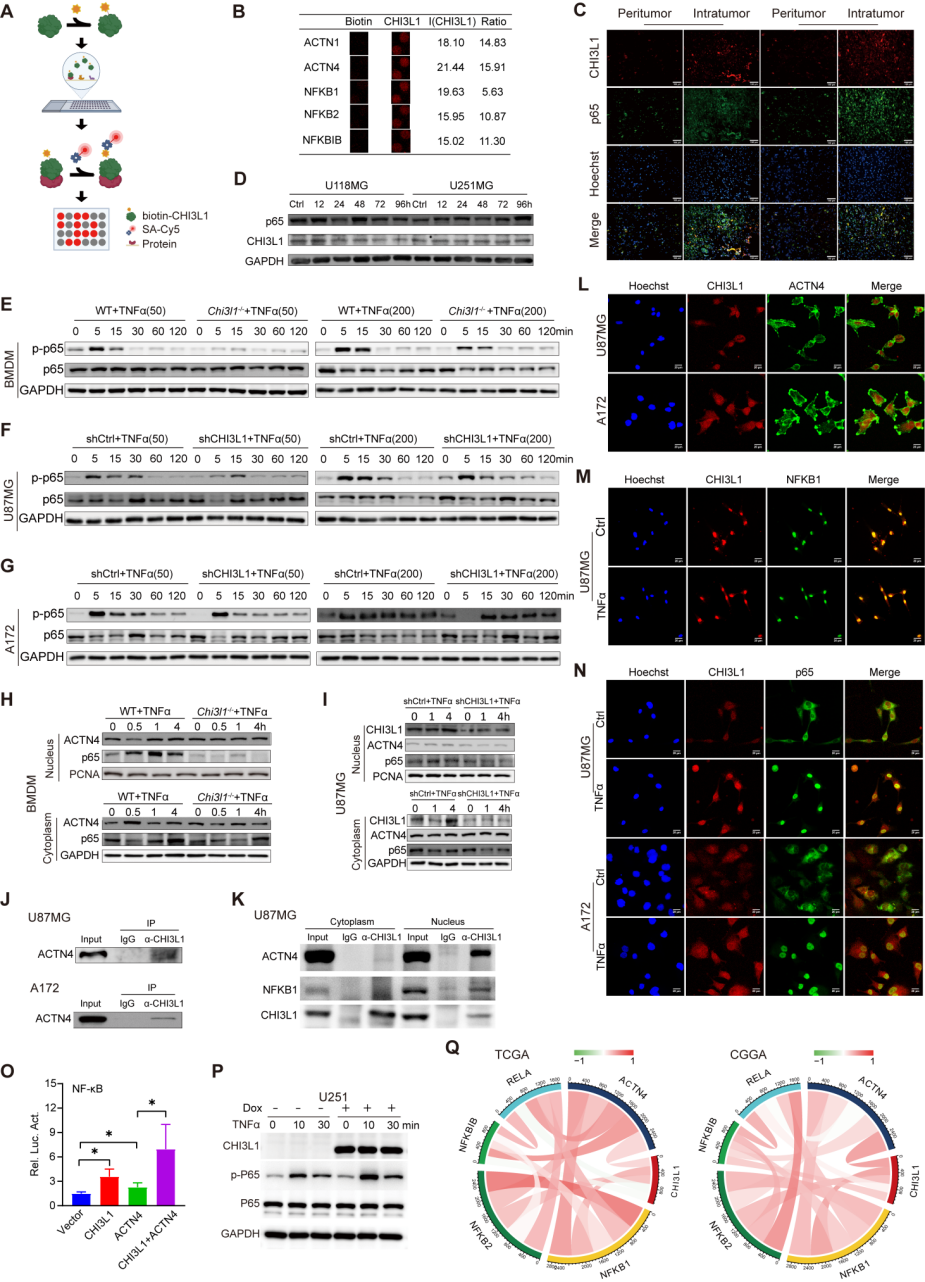
** CHI3L1 binds to ACTN4 and NFKB1, and promote the activation of NF-κB pathway**. **(A)** Schematic of the procedure used to detect biotin-hCHI3L1-binding proteins using HuProt 20K human proteome microarrays. **(B)** ACTN1, 4, NFKB1, 2, and NFKBIB were identified as CHI3L1-binding proteins in the proteome microarrays.** (C)** The IF analysis of the expression levels of CHI3L1 and NF-κB p65 subunit in peritumor and intratumor regions; Scale bars represent 100 µm. **(D)** Western blot analysis of the expression levels of CHI3L1 in U118MG and U251MG cells after treated with TNFɑ (200 ng/mL) for 0-96 h. **(E)** *Chi3l1^+/+^* and *Chi3l1^-/-^* BMDMs were treated with TNFɑ (50 and 200 ng/mL), and phosphorylation of p65 were assessed via western blot. **(F-G)** U87MG and A172 cells transfected with shCtrl and shCHI3L1 were treated with TNFɑ, and the phosphorylation of p65 were detected using western blot. **(H-I)** Cytoplasmic and nuclear proteins were isolated from BMDMs and U87MG after treated with TNFɑ (50 ng/mL) and applied to western blot to detect the expression of p65 and ACTN4. **(J)** Lysates from U87MG and A172 cells were immunoprecipitated with IgG or anti-CHI3L1 antibody, and then immunoblotted as indicated. **(K)** Cytoplasmic and nuclear proteins were isolated from U87MG cells. The lysates were immunoprecipitated with IgG or anti-CHI3L1 antibody, and then immunoblotted as indicated. **(L-N)** Co-localization of CHI3L1 and ACTN4, NFKB1, or p65 in U87MG or A172 cells observed by confocal microscope. **(O)** CHI3L1 and ACTN4 enhance NF-kB activation by using a dual-luciferase reporter assay. **(P)** Dox-inducible CHI3L1 expression enhanced enhanced NF-kB activation by TNFα in U251 cells. **(Q)** Pearson correlations between *CHI3L1* expression and *ACTN4*, *NFKB1*, *NFKB2*, *RelA* (p65), and *NFKBIB* in TCGA and CGGA glioma cohorts.

HuProt 20K human proteome microarrays containing over 21,000 affinity-purified GST-tagged proteins were used to identify CHI3L1-binding proteins (Figure 4A), and 238 proteins were identified (Supplementary Table 5). Intriguingly, ACTN1, 4, NFKB1, 2, and NFKBIB were recognized as binding partners (Figure 4B). Moreover, we performed IF staining on frozen sections of intratumor and paired peritumor tissues, and confirmed a positive correlation between CHI3L1 and NF-κB p65 expression in glioma (Figure 4C).

TNF is a major inflammatory cytokine activating the transcription factor NF-κB 23. Therefore, U118MG and U251MG cells were treated with TNFα for up to 4 days to determine whether CHI3L1 expression was dependent on sustained activation of NF-κB pathway in glioma. Western blot showed no difference between the time points in both cell lines (Figure 4D), which implied that CHI3L1 over-expression was a cause rather than a consequence of NF-κB pathway activation in glioma. We then treated WT and *Chi3l1^-/-^* BMDMs with low (50 ng/mL) and high (200 ng/mL) concentration of TNFα in the temporal gradient, and the phosphorylation of p65 subunit was obviously suppressed from five minutes in *Chi3l1^-/-^* BMDMs, both in the low and high groups (Figure 4E). Subsequently, we knocked down CHI3L1 in U87MG and A172 cells using shRNAs. Transfection and knockdown efficiencies were evaluated by fluorescence microscopy and western blot (Supplementary Figure 2A-B). Both U87MG and A172 cells transfected with shCHI3L1 displayed inhibition of p65 phosphorylation over time (Figure 4F-G).

Furthermore, we separated the nuclear and cytoplasmic proteins to explore how CHI3L1 affected the phosphorylation and nuclear translocation of NF-κB p65 subunit. Meanwhile, nuclear ACTN4 has been shown to bind to the p65 subunit of NF-κB and serve as a transcriptional co-activator to regulate transcription activity 24. Western blot revealed a clear reduction in p65 nuclear translocation in *Chi3l1^-/-^* BMDMs after treated with TNFɑ from 0.5 to 4 h, while ACTN4 showed no changes in the cytoplasm and nucleus (Figure 4H). Likewise, U87MG cells transfected with shCHI3L1 had less nuclear p65 and unchanged ACTN4 after TNFɑ treatment (Figure 4 I). Moreover, we found that CHI3L1 co-immunoprecipitated with ACTN4 in U87MG and A172 cell lines (Figure 4J). We further demonstrated that CHI3L1 bound to ACTN4 in both cytoplasm and nucleus of U87MG cells, mainly in the nucleus (Figure 4K, L). Intriguingly, it seems that CHI3L1 interacts with NFKB1 in the nucleus of U87MG cells (Figure 4K, M). In addition, the colocalization of CHI3L1 and p65 was observed in U87MG and A172 cells after TNFα stimulation (Figure 4N). Furthermore, a dual-luciferase assay demonstrated that transfection with CHI3L1 or ACTN4 enhanced the NF-kB-promoter dependent luciferase activities. Moreover, the combination of CHI3L1 and ACTN4 significantly increased the luciferase activities than ACTN4 alone (Figure 4O). In addition, we applied a dox-inducible CHI3L1 expression system in U251 cells. As shown in Figure 4P, dox-inducible CHI3L1 expression also enhanced NF-kB activation by TNFα. The Pearson correlation analysis indicated that strong positive correlations occurred between CHI3L1 and ACTN4, NFKB1 or NFKB2 in TCGA and CGGA glioma cohorts (Figure 4Q).

### CHI3L1 reprogrammed the TME to an immunosuppressive phenotype in glioma

**Figure 5:**
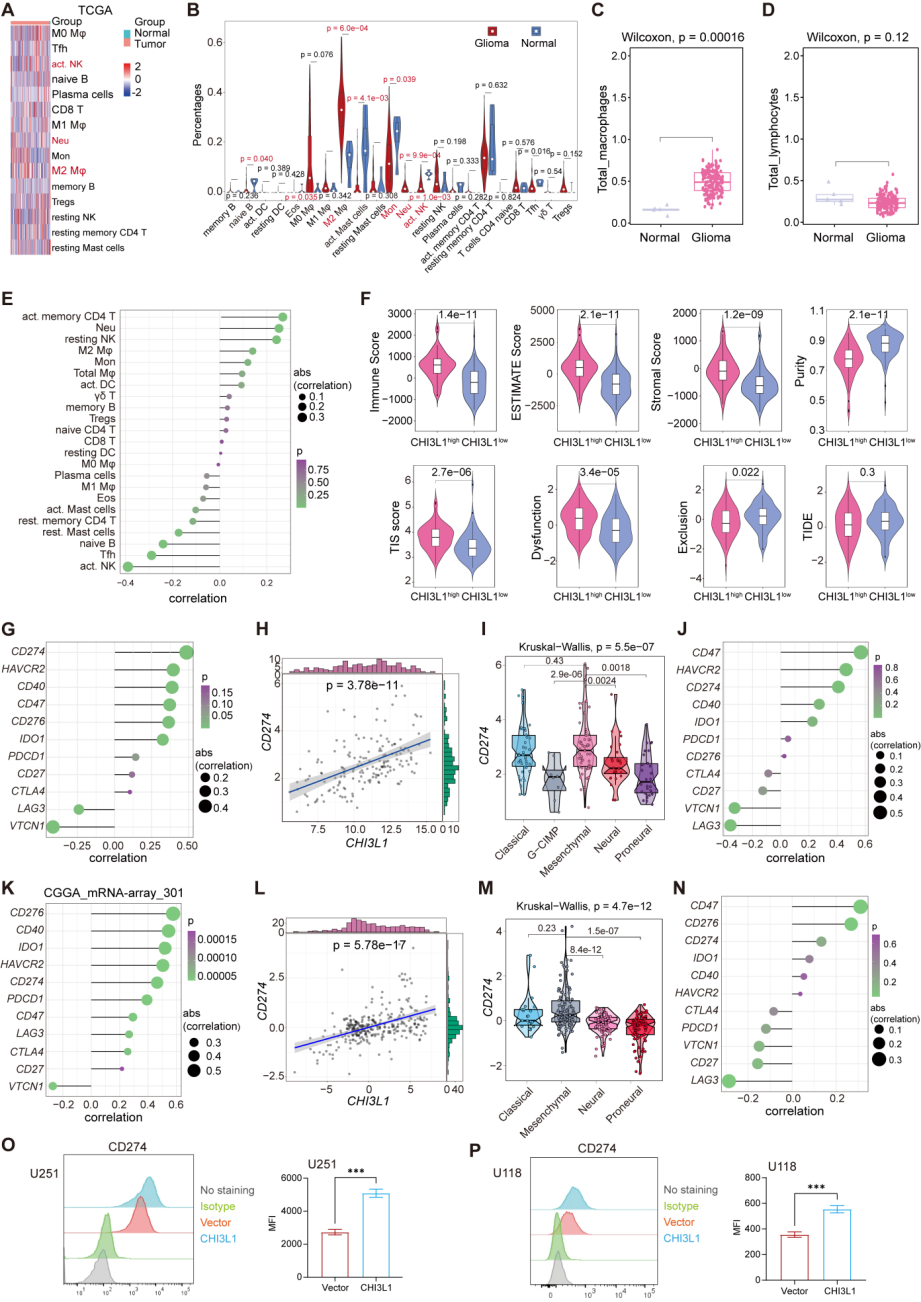
** CHI3L1 reprogrammed the TME to an immunosuppressive phenotype in glioma**. **(A-B)** Heatmap and violin plot showed the immune cells infiltration in glioma in TCGA. **(C-D)** Total macrophages and lymphocytes comparison between normal and glioma tissues in TCGA, Wilcoxon test. **(E)** Spearman correlation analysis between the CHI3L1 expression and immune cells infiltration in TCGA. **(F)** The immune score, estimate score, stromal score, tumor purity, TIS score, dysfunction, exclusion and TIDE score between the CHI3L1^high^ and CHI3L1^low^ groups of patients with glioma in TCGA, Wilcoxon test. **(G-J)** Spearman correlation analysis between the expression of CHI3L1 and immune checkpoints in glioma, as well as in MES glioma in TCGA. **(H)** *t*_student_ (163) = 7.09, *r*_Pearson_ = 0.49, CI_95%_ [0.36, 0.59], n_pairs_ = 165. log_e_ (BF_01_) = -19.44, 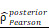
= 0.48, 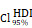 = [0.37, 0.60], 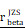 = 1.41. **(K-N)** Spearman correlation analysis between the expression of CHI3L1 and immune checkpoints in glioma, as well as in MES glioma in CGGA. **(L)** *t*_student_ (299) = 8.89, *r*_Pearson_ = 0.46, CI_95%_ [0.36, 0.54], n_pairs_ = 301. log_e_ (BF_01_) = -32.33, 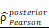
= 0.45, 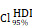 = [0.37, 0.54], 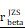 = 1.41. **(O-P)** Flow cytometry analysis of the mean fluorescence intensity (MFI) of CD274 (PDL1) staining in U251 **(O)** and U118 **(P)** cells transfected with empty vector or CHI3L1 expression plasmid. act.: activated, Neu: neutrophils, Mφ: macrophages, Mon: monocytes, DC: dendritic cells, Tregs: regulatory T cells, Eos: eosinophils, rest.: resting, Tfh: T cells follicular helper.

We inferred the absolute levels of 22 types of infiltrated immune cells within samples in TCGA cohort using Cibersort algorithm according to the LM22 signature matrix 22. We identified remarkable increases of neutrophils and M2 macrophages in tumors, while B cells naïve, monocytes, NK cells activated and T cells follicular helper showed significant decreases (Figure 5A-B). Next, the sum of M0, M1 and M2 macrophages was defined as total macrophages, and gliomas presented a higher infiltration of total macrophages than normal tissues (Figure 5C). Similarly, we classified the B and T cell subsets into total lymphocytes, and found no difference of the total lymphocytes between normal and glioma sites (Figure 5D). Spearman correlation analysis showed that CHI3L1 expression was positively correlated with the infiltration of neutrophils and macrophages M2, while NK cells activated displayed a negative correlation with the expression of CHI3L1 (Figure 5E). We then calculated the tumor immune related scores between CHI3L1^high^ and CHI3L1^low^ groups in TCGA cohort suing “ESTIMATE” R package, tumor immune dysfunction and exclusion (TIDE) portal, and 18 immune related genes 25, and found that patients with higher CHI3L1 expression exhibited a phenotype with higher tumor inflammation signature (TIS) score, immune score, estimate score, stromal score and dysfunction score, along with lower tumor purity and exclusion score (Figure 5F).

We then explored the roles of CHI3L1 in regulating the expression of immune checkpoints in glioma based on TCGA and CGGA datasets. In TCGA cohort, CHI3L1 expression was positively associated with the expression of CD274 (PD-L1) and HAVCR2 (TIM3), but had no relationship with PD-1 (Figure 5G-H). In MES glioma with the highest expression of CHI3L1, CHI3L1 expression was also associated with the level of CD274 (PD-L1) (Figure 5I-J). Consistently, the expression of CHI3L1 was correlated to CD274 (PD-L1) and HAVCR2 (TIM3) expression in CGGA database (Figure 5K-L). Likewise, MES glioma showed a higher expression of CD274 (PD-L1), which was consistent with TCGA (Figure 5M-N). Intriguingly, the expression of CD274 (PDL1) was dramatically increased in U251 and U118 cells after CHI3L1 overexpression (Figure 5O-P). Collectively, CHI3L1 altered the phenotypes and proportions of immune cells to rebuild an immunosuppressive and tumor-promoting microenvironment during the progression of glioma.

### Myeloid landscape delineation in human glioma at the single-cell level

**Figure 6:**
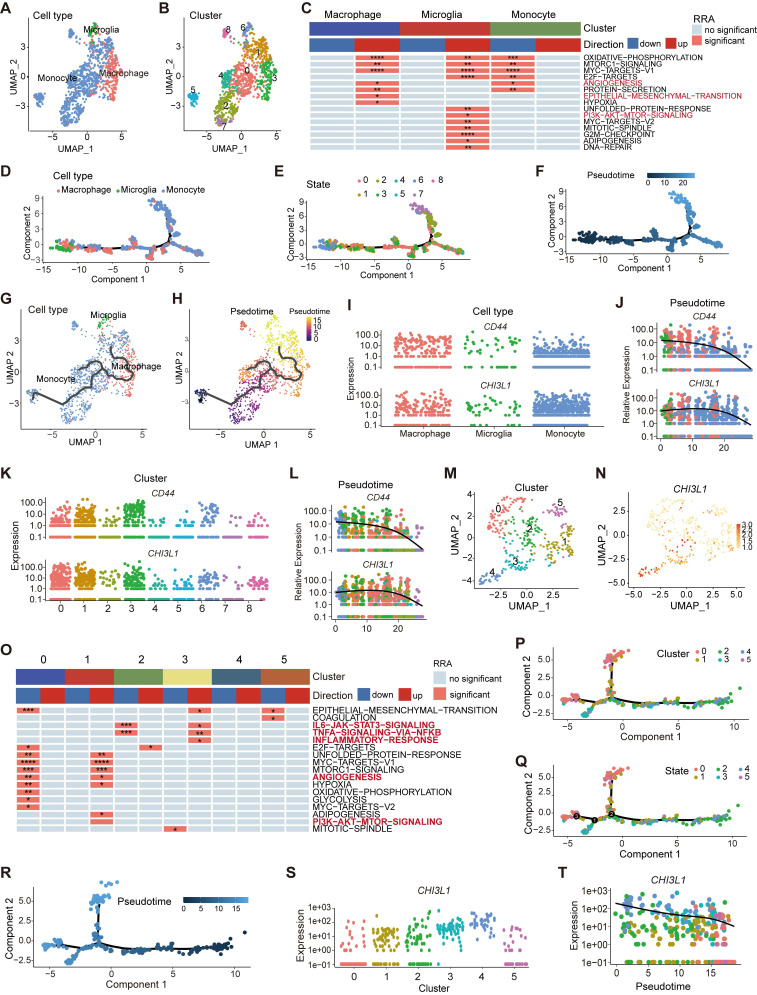
** Myeloid landscape delineation in human glioma.** UMAP plot of monocyte, microglia and macrophage populations **(A)**, with each cell color coded for cluster **(B)**. **(C)** Heatmap of hallmark gene sets with significant difference using singscore. **(D-F)** Pseudotime trajectory of monocyte, microglia, and macrophage state transition inferred by Monocle 2 and characterized by cell type **(D)**, state **(E)**, and pseudotime **(F)**. **(G-H)** Trajectory of monocytes, microglia, and macrophage state transition in UMPA plot. **(I-L)** Dynamic changes of the *CHI3L1* and *CD44* expression during the state transition profile coded for cell type **(I)**, cluster **(K)**, and pseudotime **(J, L)**.** (M)** UMAP dot plot of neutrophil from 13 patients, with each cell color coded for cluster. **(N)** Expression profile of CHI3L1 in neutrophils in the UMAP plot. **(O)** Heatmap of hallmark gene sets with significant difference in neutrophils performed by singscore. **(P-R)** Trajectory analysis of neutrophils annotated by cluster, state and pseudotime. **(S-T)** Expression profiles **(S)** and dynamic changes **(T)** of CHI3L1 among subclusters of neutrophils. *p < 0.05; **p < 0.01; ***p < 0.001; ****p < 0.0001.

To better characterize the specific phenotype and function of myeloid populations in glioma, we analyzed a public single-cell RNA-seq dataset (GSE117891) 22. We first focused on the monocyte, microglia, and macrophage axis and finally identified nine subclusters (~sc-0-8, resolution = 1) (Figure 6A-B, Supplementary Figure 3A). GSVA was applied to visualize the enriched pathway, and epithelial-mesenchymal transition (EMT) and angiogenesis pathways were significantly enriched in macrophages (Figure 6C, Supplementary Figure 3B-C). Microglia, a type of resident macrophages in the CNS 26, were enriched in PI3K AKT mTOR pathway (Figure 6C), which was an immunosuppressive target mediated by microglia 27.

TAMs in gliomas are composed of monocyte- or microglia-derived macrophages 7. Pseudo-time trajectories were appied to infer the trajectories or lineage transitions between cell types, and trajectory analysis simulated the monocyte-to-macrophage evolutionary regimes spanning different disease stages. The branched trajectory displayed two transition states at the early stages of monocyte development, and monocytes gradually differentiated into macrophages to continually replenish the population of CNS-resident macrophages (Figure 6D-F, supplementary Figure 3D). Similar to monocytes, macrophages in the brain also existed in nine different states and two origins (Figure 6E-F), implying that peripheral monocyte input was only one part of the origin of TAMs. Microglia, independent of monocyte-derived macrophages, gradually participated in the composition of TAMs as the disease progressed (Figure 6G-H), which was long considered to originate from the embryonic yolk sac 28. Interestingly, the gene expression of *CHI3L1* and *CD44* increased collaboratively in the evolution process of TAMs over pseudo-time (Figure 6I-L).

Neutrophils, another important myeloid member in the glioma TME, had been mshown to be significantly correlated with tumor grade and resistance to anti-VEGF therapy in glioma 29. We identified two populations of neutrophils by UMAP (sc-8 and 14) and divided them into six subclusters (~sc-0-5, resolution = 1) (Figure 6M, supplementary Figure 3E). CHI3L1 was predominantly expressed in sc-3 and 4, especially in sc-4 (Figure 6N). Sc-3 was significantly enriched in TNFɑ signaling via the NF-κB and IL6 JAK STAT3 signaling pathways, while sc-1 was enriched in angiogenesis and PI3K AKT mTOR signaling pathways (Figure 6O). Pseudo-time trajectories exhibited three branches at the terminal stage of developmental trajectories (Figure 6P-R), and sc-8 translated into sc-14 in terms of pseudo-time (supplementary Figure 3F-G). Notably, the expression of CHI3L1 decreased over time (Figure 6S-T).

### CHI3L1 interacts with CD44 to promote M2 macrophage polarization in TME

**Figure 7:**
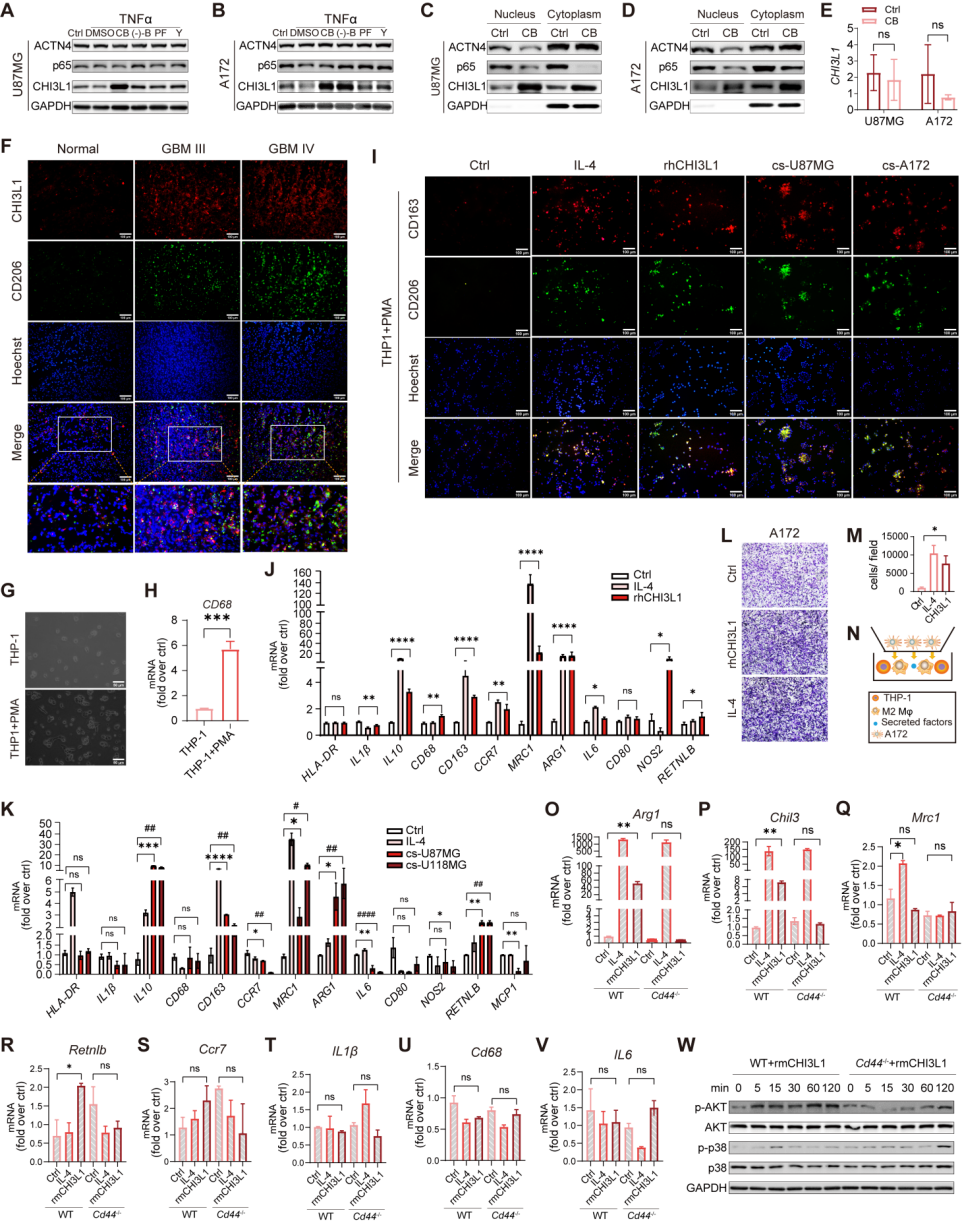
** CHI3L1 interacts with CD44 to drive M2 TAMs polarization. (A-B)** Western blot of CHI3L1, p65, and ACTN4 expression after TNFɑ treatment in U87MG and A172 cells, which were pretreated with dimethyl sulfoxide (DMSO), CB, (-)-B, PF, and Y. **(C-D)** Cytoplasmic and nuclear CHI3L1, p65, and ACTN4 were analyzed using western blot. **(E)** mRNA expression levels of CHI3L1 between the control and CB pretreated group in U87MG and A172 cell lines. **(F)** Co-immunofluorescent staining of CHI3L1 and CD206 in frozen sections of human gliomas. **(G-H)** Macrophages derived from THP1 induced by PMA (10 ng/mL) for 24 h, and identified by morphologic evaluation and mRNA expression of CD68. **(I)** M2 markers (CD206 and CD163) expression in macrophages treated with IL-4 (100 ng/mL), rhCHI3L1 (500 ng/mL), and the culture supernatant (cs) of U87MG and A172 cells measured by IF. **(J-K)** mRNA expression of M1 and M2 markers quantitated by qRT-PCR in macrophages treated with IL4, and the culture supernatant of U87MG and A172 cells. **(L-N)** Migration of U118MG cells induced by M2 macrophages. **(O-V)** mRNA expression of M1 and M2 markers in *Cd44^+/+^* and *Cd44^-/-^* BMDMs treated with IL-4 and rmCHI3L1 (500 ng/mL). **(W)** Western blot of phosphorylation of AKT and p38 in *Cd44^+/+^* and *Cd44^-/-^* BMDMs pretreated with rmCHI3L1.

Next, we investigated how CHI3L1 was secreted by glioma cells into the extracellular space. The inhibitors of filamentous actin (F-actin), microtubules, non-muscle myosin II (NM II), focal adhesion kinase (FAK), and cytoskeleton tension were used to determine the mechanical components involved 30. Correspondingly, cytochalasins B (CB), (-)-blebbistatin ((-)-B), PF573228 (PF), and Y-27632 (Y) were applied to explore the key factors controlling the secretion of CHI3L1. We found that CHI3L1 was increased in response to TNFα stimulation when pretreated with CB for 24 h, which was reported to accelerate the transformation from F-actin to globular-actin (G-actin) and decrease the content of F-actin 31. We observed no differences in the expression of ACTN4 and p65 in U87MG and A172 cell lines (Figure 7A-B). CHI3L1 expression in both the nucleus and cytoplasm was elevated in U87MG and A172 cells after treated with CB (Figure 7C-D). However, no statistically significant differences were observed in CHI3L1 expression level after CB treatment (Figure 7E). Therefore, F-actin may contribute to extracellular secretion of CHI3L1 in GBM tumor cells.

TAMs in TME are generally classified into two phenotypes: M1-like (anti-tumoral, pro-inflammatory) and M2-like (pro-tumoral, anti-inflammatory) 32. Frozen tumor and normal sections were co-immunostained for CHI3L1 and CD206 to determine the correlation between CHI3L1 distribution and M2 macrophage infiltration. We found the simultaneous high expression of CHI3L1 and CD206 were positively correlated with the tumor grade and was not co-localized within the GBM tissues (Figure 7F). We then applied a human myeloid leukemia cell line, THP-1, as a model to evaluate macrophage differentiation. THP-1 cells were differentiated into macrophages by PMA treatment for 24h, which was characterized by cell morphology and expression of the macrophage marker *CD68* (Figure 7G-H). We treated THP1 with IL-4, recombinant human CHI3L1 (rhCHI3L1), and the culture supernatants of U87-MG and A172 cells. IF analysis revealed that both rhCHI3L1 and the culture supernatant contributed to the polarization of macrophages from the M0 to M2 phenotype (Figure 7I). Meanwhile, qPCR showed the mRNA expression of M2 markers (*IL-10*, *CD163*, *MRC1*, *ARG1*, and *RETNLB*) increased markedly in macrophages after IL-4, rhCHI3L1, and culture supernatant of U87-MG and A172 cells, whereas M1 markers showed no difference or slight alterations (*HLA-DR*, *IL-1β*, *CD68*, *CD80*, *NOS2*, and *MCP-1*) (Figure 7J-K). Once differentiated into M2 phenotype by rhCHI3L1, M2 macrophages promoted the invasion of A172 cells, as observed in the transwell system (Figure 7L-N).

We previously reported that CD44v3 as a CHI3L1 receptor 33. Here, CHI3L1 and CD44 were synergistically over-expressed in macrophages at the single-cell level (Figure 6L-M). To explore the mechanisms by which CHI3L1 regulated the activation and differentiation of macrophages, we isolated BMDMs from WT and *Cd44^-/-^* mice. Consequently, *Cd44^-/-^* BMDMs treated with recombinant mouse CHI3L1 (rmCHI3L1) showed no differences of *Arg1* and *Chil3* expression compared to the control group, while rmCHI3L1 treated *Cd44^+/+^* BMDMs exhibited a significant increase (Figure 7O-P). *Mrc1*, *Retnlb*, and M1 markers (*Il-6*, *Ccr7*, *Cd68* and *IL-1β*) were not significantly changed (Figure 7Q-V). Western blot showed that AKT phosphorylation was suppressed from five minutes in rmCHI3L1 treated* Cd44^-/-^* BMDMs compared to *Cd44^+/+^* BMDMs, and p38 showed a delayed phosphorylation (Figure 7W). These results suggested CHI3L1 promoted macrophage M2 polarization through interaction with the transmembrane receptor CD44 to activate downstream PI3K/Akt signaling pathway. Indeed, Akt pathway activation had been reported to induce M2 polarization of monocyte-derived macrophages in HCV-induced hepatitis 34.

### CHI3L1 regulates the proliferation, migration, and survival of glioma cells

**Figure 8:**
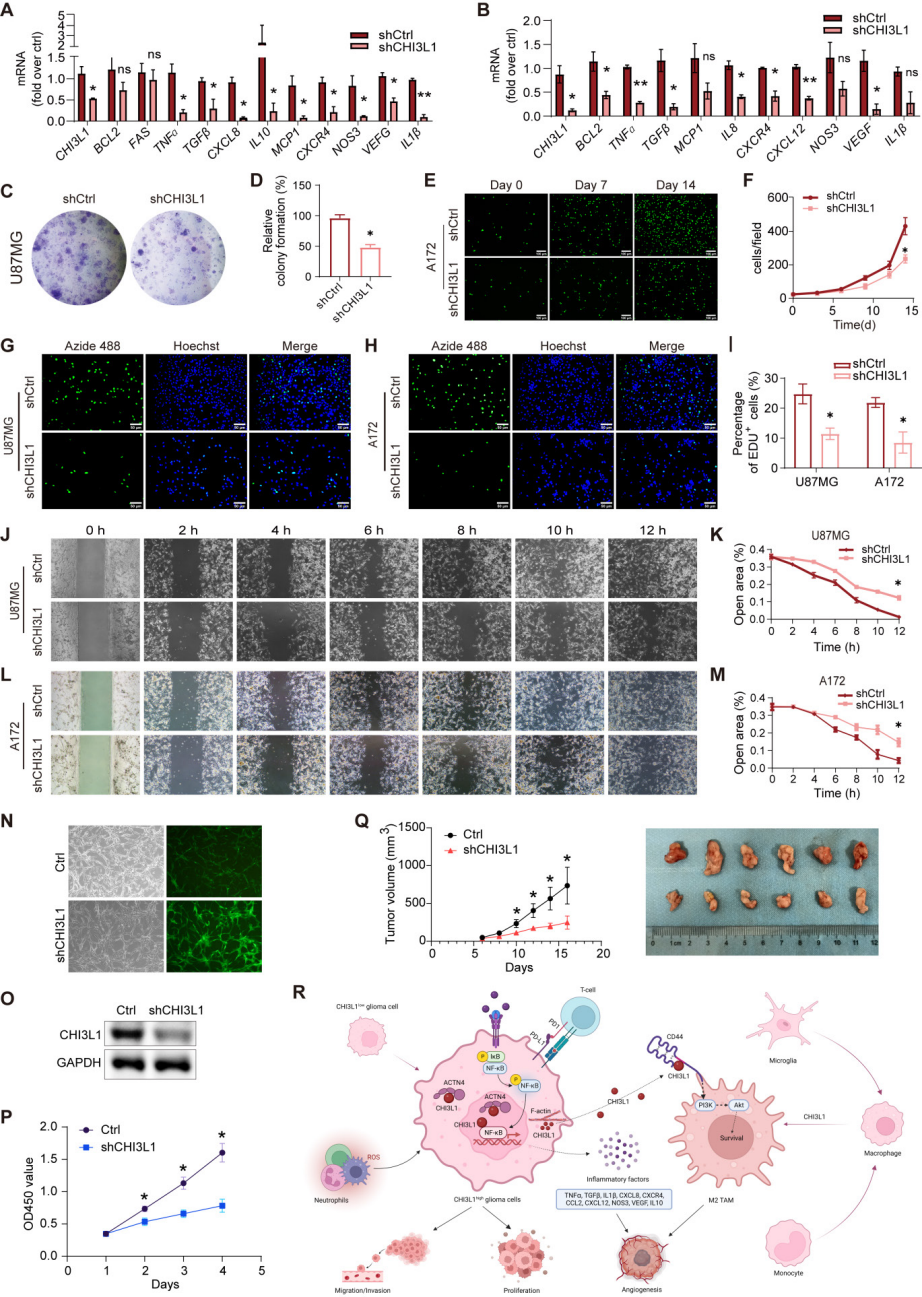
** CHI3L1 regulates the proliferation, migration and survival of glioma cells. (A-B)** The mRNA expression of inflammatory factors in U87MG and A172 cells. **(C-D)** Colony formation assay was performed in U87MG cells. **(E-F)** Proliferation curve of CFSE labelled A172 cells. **(G-I)** Proliferation of U87MG and A172 cells evaluated by EdU incorporation assay. **(J-M)** Migration of U87MG and A172 cells evaluated by wound healing assay. **(N)** The infection efficacy of the lentivirus. **(O)** The knockdown efficiency of the shCHI3L1 lentrivirus.** (P)** Proliferations of U87MG cells with or without CHI3L1 knockdown evaluated by CCK-8 assay. Student's t test, *p < 0.05, compared with the shCHI3L1 group. **(Q)** The growth rates and size of the control tumors and the tumors with CHI3L1 knockdown. Student's t test, *p < 0.05, compared with the shCHI3L1 group. **(R)** The working model of CHI3L1 on glioma cells and TME. Student's t test, *p < 0.05, *p < 0.01, compared with the shCtrl group.

Next, we found that knockdown of CHI3L1 resulted in reduced mRNA expression of inflammatory factors such as *TNFɑ*, *TGFβ*, *CXCR4*, *CXCL12*, *CCL2*, *CXCL8*, *NOS3*, and *IL1β* in U87MG and A172 cell lines (Figure 8A-B). Moreover, both U87MG and A172 cells showed decreased proliferation and survival rates due to knockdown of CHI3L1 in clonal formation (Figure 8C-F) and EdU proliferation assays (Figure 8G-I). Glioma cells with CHI3L1 knockdown also displayed reduced migration capacity, as shown by the wound healing assay (Figure 8J-M). Correspondingly, overexpression of CHI3L1 and ACTN4 enhanced the proliferation and migration of U251 or U118 cells (Supplementary Figure 4A-B). Furthermore, the U87MG cells with CHI3L1 knockdown by shCHI3L1 lentiviruses exhibited slower growth rate (Figure 8N-P). Then, the nude mice were subcutaneously injected with the U87MG cells. Consequently, the tumors developed from the cells with CHI3L1 knockdown grew slower, and the tumor sizes were also smaller than the control group (Figure 8Q). Collectively, both the *in vivo* and *in vitro* studies indicated that CHI3L1 regulates the proliferation, migration, and survival of glioma cells.

## Discussion

In the present study, we clarified that CHI3L1 was up-regulated at the mRNA level in 26 of 33 types of cancers in TCGA cohorts, especially in GBM. In fact, the expression of CHI3L1 increased with the tumor stage in both TCGA and CGGA glioma cohorts. Single-cell RNA-seq data led to the unexpected discovery that CHI3L1 was primarily expressed in glioma cells. We identified two distinct sub-populations of glioma cells with high and low expression of CHI3L1. Surprisingly, CHI3L1^high^-specific glioma cells drove the activation of the NF-κB pathway, while CHI3L1^low^ glioma cells endured a repressed state of the NF-κB pathway. All these suggested self-synthesized CHI3L1 contributed to the NF-κB signaling pathway activation in glioma cells. However, the mechanisms underlying how CHI3L1 drives NF-κB activation within tumor cells still warrant further investigation.

NF-κB activation had been reported to have a pro-proliferative function in glioma, and inhibition of this pathway presented an aggressive therapeutic approach to treat glioma 4. Knockdown of CHI3L1 resulted in decreased phosphorylation and nuclear translocation of the p65 subunit of NF-κB factor *in vitro* but had no impact on the expression of nuclear ACTN4. ACTN4, a transcriptional co-activator of the NF-κB subunit, interacts with CHI3L1 to promote the stability and transcriptional activity of NF-κB, thus regulating target genes transcription. Pseudo-time analysis of glioma populations revealed that CHI3L1^low^ glioma cells gradually converted into CHI3L1l^high^ phenotype, which was accompanied by increased enrichment score of the NF-κB pathway. Our data illustrated that CHI3L1l^low^ tumor cells gradually joined the ranks of CHI3L1l^high^ cancer cells, which initiated NF-κB activation during the progression of glioma. Continued accumulation of CHI3L1l^high^ glioma cells in the tumor foci directly resulted in sustained and enhanced activation of the NF-κB signaling pathway, which finally promoted glioma progression and malignancy.

In addition, as a secreted protein, CHI3L1 reprogrammed the TME once released into the extracellular space, which was its second felony. It has long been accepted that the inflammatory TME has a direct causative relationship with tumorigenesis 35. In the glioma TME, CHI3L1 expression positively correlated with the infiltration of M2 macrophages and neutrophils, which were acknowledged as active accomplices of glioma progression 29, 36, 37. Meanwhile, the expression of CHI3L1 was negatively associated with the number of NK cells activated, which had been demonstrated as an promising strategy for the treatment of glioma 38, 39. We also found a positive connection between the expression of CHI3L1 and immune checkpoints, such as PD-L1 (CD274) and TIM3 (HAVCR2) in TCGA and CGGA. Altogether, glioma-derived CHI3L1 remodeled the TME into a tumor-promoting and immunosuppressive profile.

Microglia- or monocyte-derived TAMs are the largest population among TME, which predominantly manifest with the M2 phenotype 40. Pseudo-time analysis of myeloid cells showed that CHI3L1 and CD44 were highly co-expressed during the gradual evolution from microglia or monocytes to macrophages. In our published research, CHI3L1 binds to CD44v3 to activate the Erk, Akt, and β-catenin pathways, thereby enhancing gastric cancer metastasis 33. Previous studies uncovered that CD44 was closely associated with the recruitment, activation, and polarization of macrophages in the TME 41. In our present study, *Cd44*^-/-^ BMDMs derived M0 macrophages failed to polarize into M2 phenotype, while *Cd44^+/+^* BMDMs successfully evolved into the M2 profile after rmCHI3L1 treatment. Paradoxically, a prior study suggested that CD44 deficiency enhances M2 polarization in non-alcoholic steatohepatitis (NASH) in mouse models 42. This discrepancy may be due to the different disease states; however, this needs to be further investigated in more detail. Further study implied that *Cd44*^-/-^ BMDMs displayed decreased phosphorylation of AKT at five minutes compared to *the Cd44^+/+^* group after rmCHI3L1 treatment, which was consistent with a published study showing that the AKT pathway was involved in macrophage polarization 43. Once evolved into M2 phenotype, M2 TAMs actively participated in the progression of glioma through angiogenesis and the EMT pathway.

Intriguingly, we demonstrated that F-actin contributed to extracellular secretion of CHI3L1 in GBM cells. Actin has two types of structural forms, F-actin and free G-actin. G-actin polymerizes to form F-actin. Two parallel F actin chains are twisted to form the double helix structure of microfilaments. In non-muscle cells, actin filaments form a track system to transport cargo such as vesicles and organelles. Recently, it was reported that Rab37 mediated CHI3L1 intracellular vesicle trafficking and exocytosis 44. Actually, F-actin and the F-actin assembly pathway regulate exocytosis 45. How F-actin regulates CHI3L1 vesicle trafficking warrants further investigations.

We elucidated in unprecedented detail how the intracellular and released CHI3L1 played pro-tumor roles in the development of glioma. We unexpectedly identified two distinct populations of neutrophils at the single-cell level, whose expression of CHI3L1 was second only to CHI3L1^high^ glioma cells. Increased infiltration of neutrophils in the TME is also positively correlated with increasing glioma malignancy 46, which may result from the overexpression of CHI3L1 to some extent. Therefore, we aim to focus on determining the roles and underlying action mechanisms of tumor-associated neutrophils in future studies.

## Conclusions

In summary, our study revealed that nuclear CHI3L1 contributed to tumor cell proliferation and survival via sustained activation of the NF-κB signaling pathway. In contrast, CHI3L1 binds to CD44 to activate the AKT pathway to facilitate M2 macrophages polarization after secreted into the TME, which is responsible for the pro-tumorigenic environment reprogramming. In conclusion, our findings provided a basis for the potential role of CHI3L1 as a therapeutic target for glioma.

## Supplementary Material

Supplementary figures and tables 1-4.

Supplementary table 5.

## Abbreviations

ACTN4: actinin alpha 4
AUC: area under the curve
BMDMs: bone-marrow-derived macrophages
CB: cytochalasins B
CGGA: Chinese Glioma Genome Atlas
CNS: central nervous system
DEGs: differentially expressed genes
ELISA: enzyme-linked immunosorbent assay
EMT: epithelial-mesenchymal transition
GBM: glioblastomas
GO: Gene Ontology
GSEA: Gene Set Enrichment Analysis
GSVA: Gene Set Variation Analysis
H&E: Hematoxylin and eosin
HGG: high grade glioma
IF: immunofluorescence
IHC: immunohistochemical
LGG: low grade glioma
MES: mesenchymal
NL: Neural
OS: overall survival
PFS: progression free survival
PL: Proneural
PMA: phorbol 12-myristate 13-acetate
rhCHI3L1: recombinant human CHI3L1
rmCHI3L1: recombinant mouse CHI3L1
TAMs: tumor-associated macrophages
TCGA: The Cancer Genome Atlas
TIDE: tumor immune dysfunction and exclusion
TIS: tumor inflammation signature
TME: tumor microenvironment
UMAP: Uniform Manifold Approximation and Projection
Y: Y-27632
(-)-B: (-)-Blebbistatin

## References

[1] Kristensen BW, Priesterbach-Ackley LP, Petersen JK, Wesseling P (2019). Molecular pathology of tumors of the central nervous system. Ann Oncol.

[2] Ostrom QT, Patil N, Cioffi G, Waite K, Kruchko C, Barnholtz-Sloan JS (2020). CBTRUS Statistical Report: Primary Brain and Other Central Nervous System Tumors Diagnosed in the United States in 2013-2017. Neuro Oncol.

[3] Zhang W, Zhangyuan G, Wang F, Jin K, Shen H, Zhang L (2021). The zinc finger protein Miz1 suppresses liver tumorigenesis by restricting hepatocyte-driven macrophage activation and inflammation. Immunity.

[4] Friedmann-Morvinski D, Narasimamurthy R, Xia Y, Myskiw C, Soda Y, Verma IM (2016). Targeting NF-kappaB in glioblastoma: A therapeutic approach. Sci Adv.

[5] Kalafati L, Kourtzelis I, Schulte-Schrepping J, Li X, Hatzioannou A, Grinenko T (2020). Innate Immune Training of Granulopoiesis Promotes Anti-tumor Activity. Cell.

[6] Chen Q, Han B, Meng X, Duan C, Yang C, Wu Z (2019). Immunogenomic analysis reveals LGALS1 contributes to the immune heterogeneity and immunosuppression in glioma. Int J Cancer.

[7] Pombo Antunes AR, Scheyltjens I, Lodi F, Messiaen J, Antoranz A, Duerinck J (2021). Single-cell profiling of myeloid cells in glioblastoma across species and disease stage reveals macrophage competition and specialization. Nat Neurosci.

[8] Nusblat LM, Carroll MJ, Roth CM (2017). Crosstalk between M2 macrophages and glioma stem cells. Cell Oncol (Dordr).

[9] Zhu C, Kros JM, Cheng C, Mustafa D (2017). The contribution of tumor-associated macrophages in glioma neo-angiogenesis and implications for anti-angiogenic strategies. Neuro Oncol.

[10] Tao W, Chu C, Zhou W, Huang Z, Zhai K, Fang X (2020). Dual Role of WISP1 in maintaining glioma stem cells and tumor-supportive macrophages in glioblastoma. Nat Commun.

[11] Felsenstein M, Blank A, Bungert AD, Mueller A, Ghori A, Kremenetskaia I (2020). CCR2 of Tumor Microenvironmental Cells Is a Relevant Modulator of Glioma Biology. Cancers (Basel).

[12] Muller S, Kohanbash G, Liu SJ, Alvarado B, Carrera D, Bhaduri A (2017). Single-cell profiling of human gliomas reveals macrophage ontogeny as a basis for regional differences in macrophage activation in the tumor microenvironment. Genome Biol.

[13] Ku BM, Lee YK, Ryu J, Jeong JY, Choi J, Eun KM (2011). CHI3L1 (YKL-40) is expressed in human gliomas and regulates the invasion, growth and survival of glioma cells. Int J Cancer.

[14] Zhao T, Su Z, Li Y, Zhang X, You Q (2020). Chitinase-3 like-protein-1 function and its role in diseases. Signal Transduct Target Ther.

[15] Yu JE, Yeo IJ, Son DJ, Yun J, Han SB, Hong JT (2021). Anti-Chi3L1 antibody suppresses lung tumor growth and metastasis through inhibition of M2 polarization. Mol Oncol.

[16] Chen Y, Zhang S, Wang Q, Zhang X (2017). Tumor-recruited M2 macrophages promote gastric and breast cancer metastasis via M2 macrophage-secreted CHI3L1 protein. J Hematol Oncol.

[17] Verhaak RG, Hoadley KA, Purdom E, Wang V, Qi Y, Wilkerson MD (2010). Integrated genomic analysis identifies clinically relevant subtypes of glioblastoma characterized by abnormalities in PDGFRA, IDH1, EGFR, and NF1. Cancer Cell.

[18] Bhat KPL, Balasubramaniyan V, Vaillant B, Ezhilarasan R, Hummelink K, Hollingsworth F (2013). Mesenchymal differentiation mediated by NF-kappaB promotes radiation resistance in glioblastoma. Cancer Cell.

[19] Newman AM, Liu CL, Green MR, Gentles AJ, Feng W, Xu Y (2015). Robust enumeration of cell subsets from tissue expression profiles. Nat Methods.

[20] Wang Y, Weng X, Wang L, Hao M, Li Y, Hou L (2018). HIC1 deletion promotes breast cancer progression by activating tumor cell/fibroblast crosstalk. J Clin Invest.

[21] Chandra A, Jahangiri A, Chen W, Nguyen AT, Yagnik G, Pereira MP (2020). Clonal ZEB1-Driven Mesenchymal Transition Promotes Targetable Oncologic Antiangiogenic Therapy Resistance. Cancer Res.

[22] Yu K, Hu Y, Wu F, Guo Q, Qian Z, Hu W (2020). Surveying brain tumor heterogeneity by single-cell RNA-sequencing of multi-sector biopsies. Natl Sci Rev.

[23] Van Quickelberghe E, De Sutter D, van Loo G, Eyckerman S, Gevaert K (2018). A protein-protein interaction map of the TNF-induced NF-kappaB signal transduction pathway. Scientific data.

[24] Lomert E, Turoverova L, Kriger D, Aksenov ND, Nikotina AD, Petukhov A (2018). Co-expression of RelA/p65 and ACTN4 induces apoptosis in non-small lung carcinoma cells. Cell Cycle.

[25] Ayers M, Lunceford J, Nebozhyn M, Murphy E, Loboda A, Kaufman DR (2017). IFN-gamma-related mRNA profile predicts clinical response to PD-1 blockade. J Clin Invest.

[26] Muffat J, Li Y, Yuan B, Mitalipova M, Omer A, Corcoran S (2016). Efficient derivation of microglia-like cells from human pluripotent stem cells. Nat Med.

[27] Dumas AA, Pomella N, Rosser G, Guglielmi L, Vinel C, Millner TO (2020). Microglia promote glioblastoma via mTOR-mediated immunosuppression of the tumour microenvironment. EMBO J.

[28] Leid J, Carrelha J, Boukarabila H, Epelman S, Jacobsen SE, Lavine KJ (2016). Primitive Embryonic Macrophages are Required for Coronary Development and Maturation. Circ Res.

[29] Liang J, Piao Y, Holmes L, Fuller GN, Henry V, Tiao N (2014). Neutrophils promote the malignant glioma phenotype through S100A4. Clin Cancer Res.

[30] Liu Z, Wang L, Xu H, Du Q, Li L, Wang L (2020). Heterogeneous Responses to Mechanical Force of Prostate Cancer Cells Inducing Different Metastasis Patterns. Advanced science.

[31] Zhou C, Wang Y, Pan D, Sun Y, Cao J (2016). The effect of Cytochalasin B and Jasplakinolide on depolymerization of actin filaments in goose muscles during postmortem conditioning. Food Res Int.

[32] Liu C, Chikina M, Deshpande R, Menk AV, Wang T, Tabib T (2019). Treg Cells Promote the SREBP1-Dependent Metabolic Fitness of Tumor-Promoting Macrophages via Repression of CD8(+) T Cell-Derived Interferon-gamma. Immunity.

[33] Geng B, Pan J, Zhao T, Ji J, Zhang C, Che Y (2018). Chitinase 3-like 1-CD44 interaction promotes metastasis and epithelial-to-mesenchymal transition through beta-catenin/Erk/Akt signaling in gastric cancer. J Exp Clin Cancer Res.

[34] Kwon YC, Meyer K, Peng G, Chatterjee S, Hoft DF, Ray R (2019). Hepatitis C Virus E2 Envelope Glycoprotein Induces an Immunoregulatory Phenotype in Macrophages. Hepatology.

[35] Venteicher AS, Tirosh I, Hebert C, Yizhak K, Neftel C, Filbin MG (2017). Decoupling genetics, lineages, and microenvironment in IDH-mutant gliomas by single-cell RNA-seq. Science.

[36] Wu J, Frady LN, Bash RE, Cohen SM, Schorzman AN, Su YT (2018). MerTK as a therapeutic target in glioblastoma. Neuro Oncol.

[37] Meng X, Duan C, Pang H, Chen Q, Han B, Zha C (2019). DNA damage repair alterations modulate M2 polarization of microglia to remodel the tumor microenvironment via the p53-mediated MDK expression in glioma. EBioMedicine.

[38] Han J, Chen X, Chu J, Xu B, Meisen WH, Chen L (2015). TGFbeta Treatment Enhances Glioblastoma Virotherapy by Inhibiting the Innate Immune Response. Cancer Res.

[39] Wu P, Geng B, Chen Q, Zhao E, Liu J, Sun C (2020). Tumor Cell-Derived TGFbeta1 Attenuates Antitumor Immune Activity of T Cells via Regulation of PD-1 mRNA. Cancer immunology research.

[40] Wang Q, Hu B, Hu X, Kim H, Squatrito M, Scarpace L (2017). Tumor Evolution of Glioma-Intrinsic Gene Expression Subtypes Associates with Immunological Changes in the Microenvironment. Cancer Cell.

[41] Hossaini Nasr S, Rashidijahanabad Z, Ramadan S, Kauffman N, Parameswaran N, Zinn KR (2020). Effective atherosclerotic plaque inflammation inhibition with targeted drug delivery by hyaluronan conjugated atorvastatin nanoparticles. Nanoscale.

[42] Patouraux S, Rousseau D, Bonnafous S, Lebeaupin C, Luci C, Canivet CM (2017). CD44 is a key player in non-alcoholic steatohepatitis. J Hepatol.

[43] Vergadi E, Ieronymaki E, Lyroni K, Vaporidi K, Tsatsanis C (2017). Akt Signaling Pathway in Macrophage Activation and M1/M2 Polarization. J Immunol.

[44] Yang PS, Yu MH, Hou YC, Chang CP, Lin SC, Kuo IY (2022). Targeting protumor factor chitinase-3-like-1 secreted by Rab37 vesicles for cancer immunotherapy. Theranostics.

[45] Wu LG, Chan CY (2022). Multiple Roles of Actin in Exo- and Endocytosis. Frontiers in synaptic neuroscience.

[46] Kan LK, Seneviratne S, Drummond KJ, Williams DA, O'Brien TJ, Monif M (2020). P2X7 receptor antagonism inhibits tumour growth in human high-grade gliomas. Purinergic Signal.

