# Supplementary material for: Chitinase-3 like-protein-1 promotes glioma progression via the NF-κB signaling pathway and tumor microenvironment reprogramming
Source: Theranostics. 2022 Oct 3;12(16):6989–7008. doi: 10.7150/thno.75069 (PMC9576612; doi:10.7150/thno.75069)
Supplement: Supplementary file 1 — Supplementary figures and tables 1-4. [file thnov12p6989s1.pdf]

Supplementary Figure legends

Supplementary Figure 1. Magnetic resonance imaging (MRI) and 3D model reconstruction-guided tumor localization and surgical sampling.

Supplementary Figure 2. Knockdown of CHI3L1 in U87MG and A172 cell lines. Transfection and knockdown efficiencies were validated by fluorescence microscopy (A) and western blot (B), respectively; Scale bars represent 100 μm.

Supplementary Figure 3. Myeloid landscape delineation in human glioma. (A) UMAP plot of monocyte, microglia and macrophage populations, with each cell color coded for cell type. **(B-C)** The distribution of enrichment score of EMT **(B)** and angiogenesis **(C)** pathway in UMAP plot. **(D)** Pseudotime trajectory of monocyte, microglia, and macrophage state transition inferred by Monocle 2 and characterized by cell type. **(E)** UMAP dot plot of neutrophil from 13 patients, with each cell color coded for cell type. **(F-G)** UMAP plot showed the evolutionary trajectory of neutrophils by cluster **(F)** and pseudotime **(G)**.

Supplementary Figure 4. Overexpression of CHI3L1 or ACTN4 promotes glioma cells proliferation and migration. A, Proliferation of U251 cells evaluated by CCK-8 assay. B. Migration of U251 and U118 cells evaluated by wound healing assay; Student's t test, $^{*}p < 0.05$, compared with the control group; $^{\#}p < 0.05$, compared with the CHI3L1 group; $^{\&}p < 0.05$, compared with the ACTN4 group.

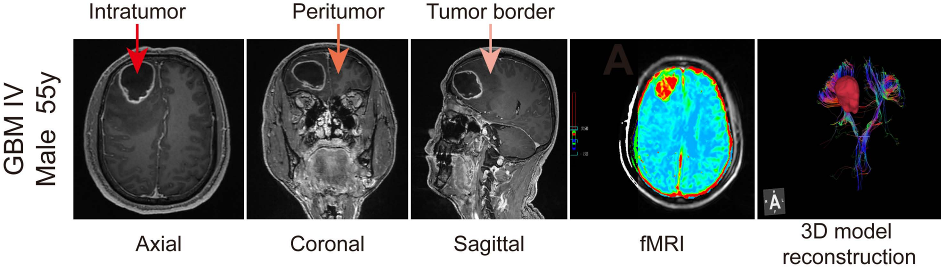

Intratumor
Peritumor
Tumor border
GBM IV
Male 55y
Axial
Coronal
Sagittal
fMRI
3D model reconstruction

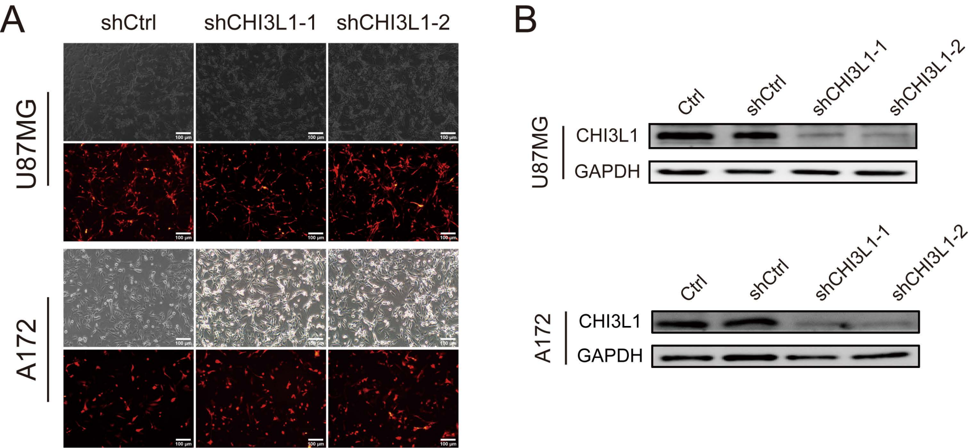
A
shCtrl
shCHI3L1-1
shCHI3L1-2
U87MG
A172
100 μm
B
Ctrl
shCtrl
shCHI3L1-1
shCHI3L1-2
U87MG
CHI3L1
GAPDH
A172
CHI3L1
GAPDH

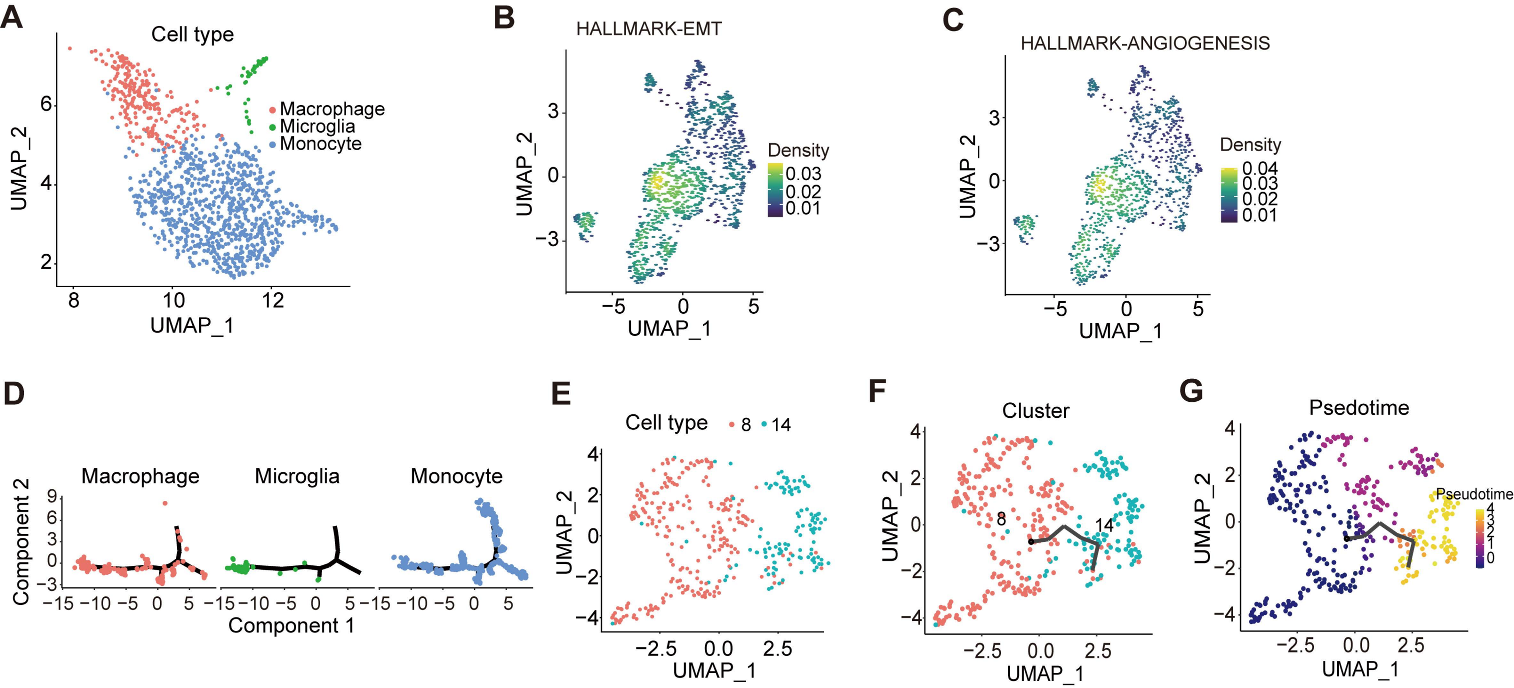

A
Cell type
Macrophage
Microglia
Monocyte
UMAP_1
UMAP_2
B
HALLMARK-EMT
Density
C
HALLMARK-ANGIOGENESIS
Density
D
Macrophage
Microglia
Monocyte
Component 1
Component 2
E
Cell type
8
14
F
Cluster
G
Psedotime
Pseudotime

A

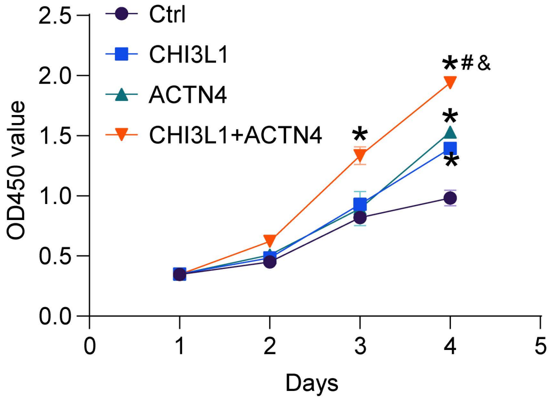

Ctrl
CHI3L1
ACTN4
CHI3L1+ACTN4
OD450 value
Days
2.5
2.0
1.5
1.0
0.5
0.0
0
1
2
3
4
5
*#&
*
*
*

B

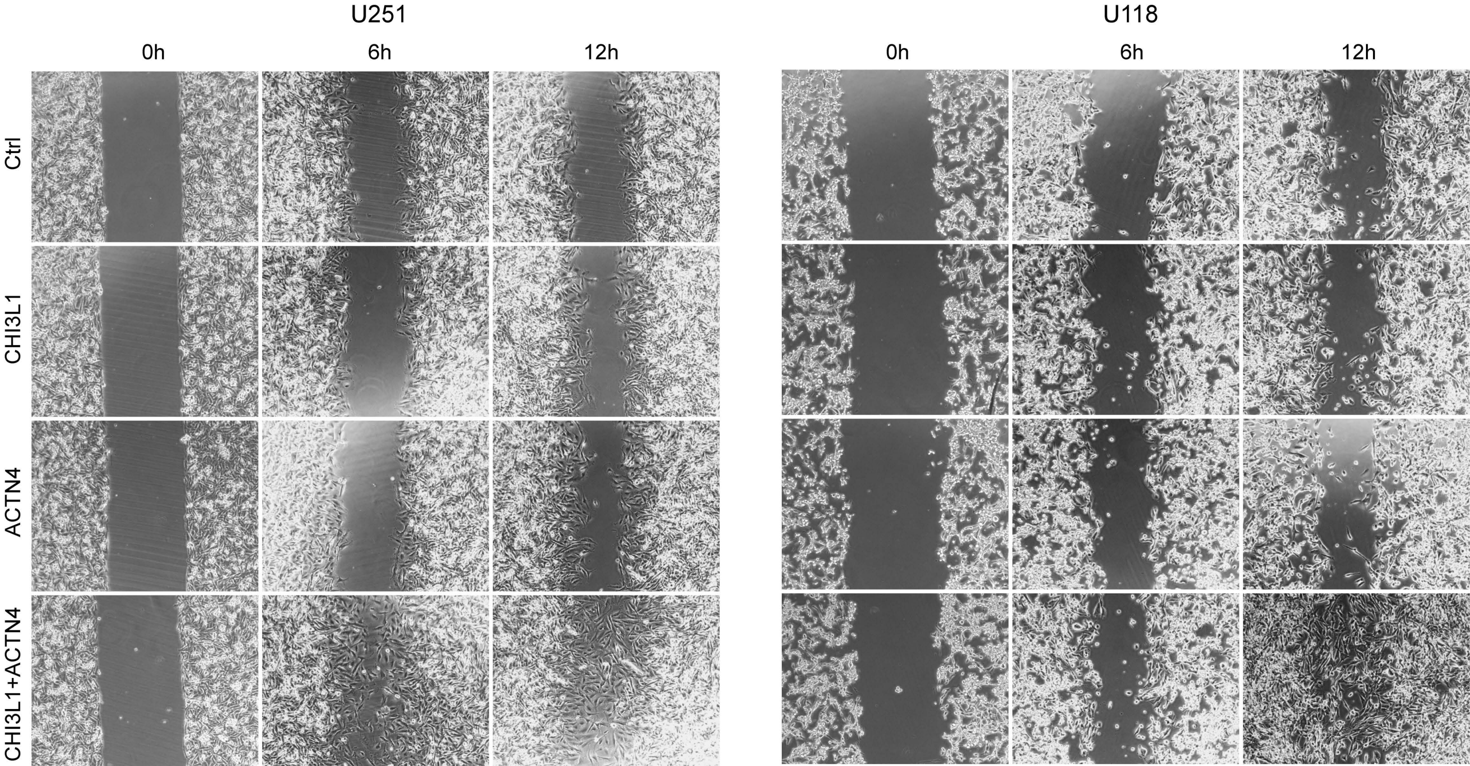

U251
U118
0h
6h
12h
0h
6h
12h
Ctrl
CHI3L1
ACTN4
CHI3L1+ACTN4

Supplementary table 1: Summary of antibodies applied in western blot, related to section "Western Blot".

Name	Manufacturer	Cat.	Dilution rate
GAPDH	CST, USA	5174S	1:1000
CHI3L1	Abcam, USA	ab77528	1:1000
Phospho-NF-κB p65	CST, USA	3033S	1:1000
NF-κB p65	CST, USA	6956S	1:1000
NFKB1	Proteintech, China	66992-1-Ig	1:1000
Phospho-SAPK/JNK	CST, USA	9251S	1:1000
JNK2	CST, USA	9258S	1:1000
Phospho-p44/42 MAPK (Erk1/2)	CST, USA	4370S	1:1000
ERK1 + ERK2	Abcam, USA	ab184699	1:1000
Phospho-Akt (Ser473)	CST, USA	4060S	1:1000
Akt	CST, USA	4691S	1:1000
Phospho-p38 MAPK (Thr180/Tyr182)	CST, USA	4511S	1:1000
p38α MAPK	CST, USA	9218S	1:1000
PCNA	CST, USA	2586S	1:1000
α-actinin-4	Santa Cruz, USA	sc-393495	1:100

Supplementary table 2: Summary of primer sequence, related to section “Quantitative real-time PCR (qRT-PCR)”.

Gene name		Sequence
Homo-GAPDH	Forward Primer	GTCTCCTCTGACTTCAACAGCG
	Reverse Primer	ACCACCCTGTTGCTGTAGCCAA
Homo-CD163	Forward Primer	CCAGAAGGAACTTGTAGCCACAG
	Reverse Primer	CAGGCACCAAGCGTTTTGAGCT
Homo-IL-10	Forward Primer	TCTCCGAGATGCCTTCAGCAGA
	Reverse Primer	TCAGACAAGGCTTGGCAACCCA
Homo-CD68	Forward Primer	CGAGCATCATTCTTTCACCAGCT
	Reverse Primer	ATGAGAGGCAGCAAGATGGACC
Homo-Arg1	Forward Primer	TCATCTGGGTGGATGCTCACAC
	Reverse Primer	GAGAATCCTGGCACATCGGGAA
Homo-RETNLB	Forward Primer	GCAAGAAGCTCTCGTGTGCTAG
	Reverse Primer	AACATCCCACGAACCACAGCCA
Homo-IL-1β	Forward Primer	CCACAGACCTTCCAGGAGAATG
	Reverse Primer	GTGCAGTTCAGTGATCGTACAGG
Homo-CD197	Forward Primer	CAACATCACCAGTAGCACCTGTG
	Reverse Primer	TGCGGAACTTGACGCCGATGAA
Homo-HLA-DR	Forward Primer	AGCTGTGGACAAAGCCAACCTG
	Reverse Primer	CTCTCAGTTCCACAGGGCTGTT
Homo-IL-6	Forward Primer	AGACAGCCACTCACCTCTTCAG
	Reverse Primer	TTCTGCCAGTGCCTCTTTGCTG
Homo-MCP-1	Forward Primer	AGAATCACCAGCAGCAAGTGTCC
	Reverse Primer	TCCTGAACCCACTTCTGCTTGG
Homo-iNOS	Forward Primer	GCTCTACACCTCCAATGTGACC
	Reverse Primer	CTGCCGAGATTTGAGCCTCATG
Homo-eNOS	Forward Primer	GAAGGCGACAATCCTGTATGGC
	Reverse Primer	TGTTCGAGGGACACCACGTCAT

Homo-CXCL12	Forward Primer	CTCAACACTCCAAACTGTGCCC
	Reverse Primer	CTCCAGGTACTCCTGAATCCAC
Homo-CXCR4	Forward Primer	CTCCTCTTTGTCATCACGCTTCC
	Reverse Primer	GGATGAGGACACTGCTGTAGAG
Homo-VEGF	Forward Primer	TTGCCTTGCTGCTCTACCTCCA
	Reverse Primer	GATGGCAGTAGCTGCGCTGATA
Mus-CD206	Forward Primer	GTTCACCTGGAGTGATGGTTCTC
	Reverse Primer	AGGACATGCCAGGGTCACCTTT
Mus-CD163	Forward Primer	GGCTAGACGAAGTCATCTGCAC
	Reverse Primer	CTTCGTTGGTCAGCCTCAGAGA
Mus-CD68	Forward Primer	GGCGGTGGAATACAATGTGTCC
	Reverse Primer	AGCAGGTCAAGGTGAACAGCTG
Mus-RETNLB	Forward Primer	GAACGCGCAATGCTCCTTTGAG
	Reverse Primer	AGCCACAAGCACATCCAGTGAC
Mus-CD197	Forward Primer	AGAGGCTCAAGACCATGACGGA
	Reverse Primer	TCCAGGACTTGGCTTCGCTGTA
Mus-CD80	Forward Primer	CCTCAAGTTTCCATGTCCAAGGC
	Reverse Primer	GAGGAGAGTTGTAACGGCAAGG
Mus-Ym1	Forward Primer	TACTCACTTCCACAGGAGCAGG
	Reverse Primer	CTCCAGTGTAGCCATCCTTAGG

Supplementary table 3. Correlation between mRNA expression of CHI3L1 and clinical characteristics in patients with glioma in TCGA and CCGA (mRNA-array_301) dataset. R/S: Recurrent/Secondary

Characteristics	Total (N)	CGGA_ mRNA-array_301			Total (N)	TCGA		
		$CHI3L1^{high}$	$CHI3L1^{low}$	p value		$CHI3L1^{high}$	$CHI3L1^{low}$	p value
PRS_type				0.0684				0.1774
Primary	264 (88.6%)	127 (42.6%)	137(46.0%)		160 (97.0%)	79 (47.9%)	81 (49.1%)	
R/S	34 (11.4%)	22 (7.4%)	12 (4.0%)		5 (3.0%)	4 (2.4%)	1 (0.6%)	
Age				**0.0002**				0.1616
<=60	276 (92.3%)	130 (38.1%)	146 (54.2%)		78 (47.5%)	35 (21.3%)	43 (26.2%)	
>60	23 (7.7%)	20 (5.7%)	3 (2.0%)		86 (52.5%)	48 (29.3%)	38 (23.2%)	
Gender				0.8691				0.0677
Male	180 (59.8%)	91 (30.2%)	89 (29.6%)		104 (63.4%)	47 (30.5%)	57 (32.9%)	
Female	121 (40.2%)	60 (19.9%)	61 (20.3%)		60 (36.6%)	36 (22.6%)	24 (14.0%)	
Grade				**<0.0001**				/
II	117 (39.3%)	17 (5.7%)	100 (33.6%)		/	/	/	
III	57 (19.1%)	30 (10.1%)	27 (9.0%)		/	/	/	
IV	124 (41.6%)	102 (34.2%)	22 (7.4%)		/	/	/	
TCGA subtype				**<0.0001**				**<0.0001**
MES	111 (36.9%)	99 (32.9%)	12 (4.0%)		56 (34.0%)	43 (26.1%)	13 (7.9%)	
CL	23 (7.6%)	16 (5.3%)	7 (2.3%)		42 (25.4%)	19 (11.5%)	23 (13.9%)	
NL	81 (26.9%)	18 (6.0%)	63 (20.9%)		28 (17.0%)	16 (9.7%)	12 (7.3%)	
PL	86 (28.6%)	18 (6.0%)	68 (22.6%)		31 (18.8%)	5 (3.0%)	26 (15.8%)	
G-CIMP	/	/	/		8 (4.8%)	0 (0.0%)	8 (4.8%)	
Radio status				0.6446				0.4141
untreated	46 (16.3%)	21 (7.5%)	25 (8.8%)		6 (3.6%)	4 (2.4%)	2 (1.2%)	
treated	237 (83.7%)	117 (41.3%)	120 (42.4%)		159 (96.4%)	79 (50.3%)	80 (46.1%)	
TMZ status				**0.0014**				0.2709
untreated	144 (52.0%)	60 (21.7%)	84 (30.3%)		55 (33.3%)	31 (18.8%)	24 (14.5%)	
treated	133 (48.0%)	81 (29.2%)	52 (18.8%)		110 (66.7%)	52 (31.5%)	58 (35.2%)	
IDH status				**<0.0001**				**0.0015**
WT	165 (55.2%)	123 (41.1%)	42 (14.1%)		151 (94.4%)	82 (51.3%)	69 (43.1%)	
Mut	134 (44.8%)	28 (9.4%)	106 (35.4%)		9 (5.6%)	0 (0.0%)	9 (5.6%)	
1p19q codeletion				**<0.0001**				
non-codel	76 (82.6%)	45 (48.9%)	31 (33.7%)		/	/	/	
codel	16 (17.4%)	0 (0.0%)	16 (17.4%)		/	/	/	

Characteristics	Total (N)	CGGA_ mRNA-array_301			Total (N)	TCGA		
		CHI3L1^{high}	CHI3L1^{low}	p value		CHI3L1^{high}	CHI3L1^{low}	p value
MGMTp methylation				0.3438				**0.0274**
unmethylated	187 (65.4%)	91 (31.8%)	96 (33.6%)		68 (54.0%)	38 (30.2%)	30 (23.8%)	
methylated	99 (34.6%)	54 (18.9%)	45 (15.7%)		58 (46.0%)	21(16.6%)	37 (29.4%)	
G-CIMP methylation				/				**0.0018**
unmethylated	/	/	/		155 (94.5%)	83 (50.6%)	72 (43.9%)	
methylated	/	/	/		9 (5.5%)	0 (0.0%)	9 (5.5%)	
Censor				**<0.0001**				0.1441
Living	100 (34.8%)	25 (8.7%)	75 (26.1%)		50 (30.5%)	21 (12.8%)	29 (17.7%)	
Deceased	187 (65.2%)	123 (42.9%)	64 (22.3%)		114 (69.5%)	62 (37.8%)	52 (31.7%)	

Supplementary table 4. Univariate Cox regression analysis of clinical characteristics associated with overall survival in CCGA mRNA-array_301 and CGGA mRNAseq_693 dataset.

Characteristics	Total (N)	CGGA_mRNA-array_301		Total (N)	CGGA_mRNAseq_693	
		Hazard ratio (95%CI)	p value		Hazard ratio (95%CI)	p value
CHI3L1	301			693		
Low		Reference			Reference	
High		3.949 (2.882-5.413)	**<2e-16**		3.184 (2.578-3.932)	**<2e-16**
TCGA subtypes	301			/		
MES		Reference			/	
CL		0.891 (0.523-1.519)	0.6720		/	/
NL		0.309 (0.206-0.463)	**1.25e-08**		/	/
PL		0.386 (0.269-0.552)	**1.99e-07**		/	/
PRS	298			693		
Primary		Reference			Reference	
Recurrent/secondary		2.369 (1.546-3.631)	**7.43e-05**		2.182 (1.785-2.667)	**2.57e-14**
Grade	298			692		
II		Reference			Reference	
III		2.990 (1.892-4.725)	**2.74e-06**		2.545 (1.846-3.509)	**1.17e-08**
IV		6.938 (4.725-10.187)	**< 2e-16**		6.972 (5.085-9.561)	**< 2e-16**
Gender	301			693		
Female		Reference			Reference	
Male		1.280 (0.950-1.723)	0.104		1.061 (0.868-1.297)	0.563
Age	299			692		
<=60		Reference			Reference	
>60		2.236 (1.403-3.565)	**0.000716**		2.225 (1.681-2.944)	**2.24e-08**
Radio status	283			646		
Untreated		Reference			Reference	
Treated		0.569 (0.384-0.844)	**0.00501**		1.241 (0.953-1.615)	0.1090
TMZ status	277			647		
Untreated		Reference			Reference	
Treated		1.126 (0.839-1.513)	0.429		1.243 (0.974-1.586)	0.0809

Characteristics	Total (N)	CGGA_mRNA-array_301		Total (N)	CGGA_mRNAseq_693	
		Hazard ratio (95%CI)	p value		Hazard ratio (95%CI)	p value
IDH status	301			642		
Wildtype		Reference			Reference	
Mutant		0.391 (0.287-0.533)	**2.46e-09**		0.323 (0.262-0.398)	**<2e-16**
1p19q codeletion	92			623		
Non-codel		Reference			Reference	
Codel		0.149 (0.053-0.416)	**0.000274**		0.268 (0.193-0.372)	**2.98e-15**
MGMTp methylation	286			542		
Unmethylated		Reference			Reference	
Methylated		1.234 (0.912-1.670)	0.173		0.795 (0.639-0.990)	**0.0407**
